# Defect {(W^VI^O_7_)W^VI^_4_} and Full {(W^VI^O_7_)W^VI^_5_} Pentagonal Units as Synthons for the Generation of Nanosized Main Group V Heteropolyoxotungstates

**DOI:** 10.1021/acs.inorgchem.1c00810

**Published:** 2021-06-04

**Authors:** Elias Tanuhadi, Nadiia I. Gumerova, Alexander Prado-Roller, Andreas Mautner, Annette Rompel

**Affiliations:** † Universität Wien, Fakultät für Chemie, Institut für Biophysikalische Chemie, 1090 Wien, Austria; ‡ Universität Wien, Fakultät für Chemie, Zentrum für Röntgenstrukturanalyse und Institut für Anorganische Chemie, 1090 Wien, Austria; ⊥ Universität Wien, Fakultät für Chemie, Polymer and Composite Engineering (PaCE) Group, Institute of Materials Chemistry and Research, 1090 Vienna, Austria

## Abstract

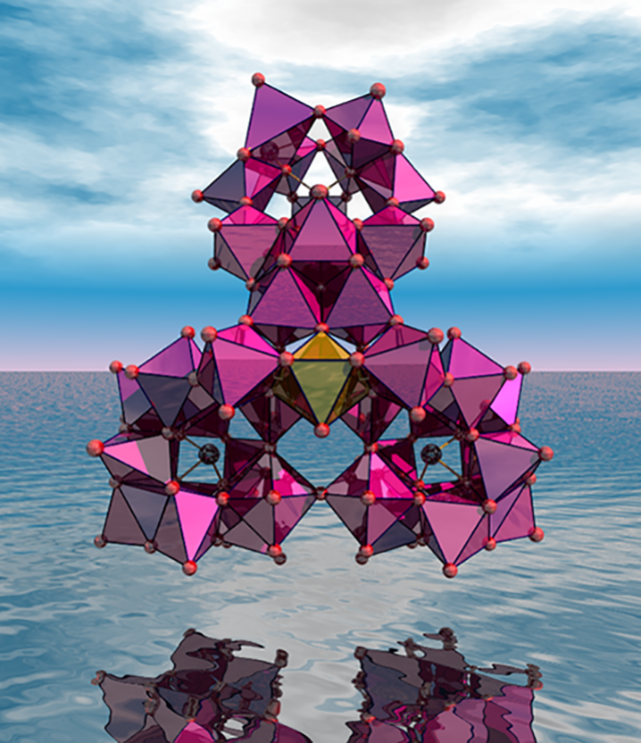

We report on the synthesis and characterization of three new nanosized main group V heteropolyoxotungstates K_*x*_Na_*y*_[H_2_(XW^VI^_9_O_33_)(W^VI^_5_O_12_)(X_2_W^VI^_29_O_103_)]·*n*H_2_O **{X**_**3**_**W**_**43**_**}** (*x* = 11, *y* = 16, and *n* = 115.5 for X = Sb^III^; *x* = 20, *y* = 7, and *n* = 68 for X = Bi^III^) and K_8_Na_15_[H_16_(Co^II^(H_2_O)_2_)_0.9_(Co^II^(H_2_O)_3_)_2_(W^VI^_3.1_O_14_)(Sb^III^W^VI^_9_O_33_)(Sb^III^_2_W^VI^_30_O_106_)(H_2_O)]·53H_2_O **{Co**_**3**_**Sb**_**3**_**W**_**42**_**}**. On the basis of the key parameters for the one-pot synthesis strategy of **{Bi**_**3**_**W**_**43**_**}**, a rational step-by-step approach was developed using the known Krebs-type polyoxotungstate (POT) K_12_[Sb^V^_2_W^VI^_22_O_74_(OH)_2_]·27H_2_O **{Sb**_**2**_**W**_**22**_**}** as a nonlacunary precursor leading to the synthesis and characterization of **{Sb**_**3**_**W**_**43**_**}** and **{Co**_**3**_**Sb**_**3**_**W**_**42**_**}**. Solid-state characterization of the three new representatives **{Bi**_**3**_**W**_**43**_**}**, **{Sb**_**3**_**W**_**43**_**}**, and **{Co**_**3**_**Sb**_**3**_**W**_**42**_**}** by single-crystal and powder X-ray diffraction (XRD), IR spectroscopy, thermogravimetric analysis (TGA), energy-dispersive X-ray analysis (EDX), X-ray photoelectron spectroscopy (XPS), and elemental analysis, along with characterization in solution by UV/vis spectroscopy shows that **{Bi**_**3**_**W**_**43**_**}**, **{Sb**_**3**_**W**_**43**_**}**, and **{Co**_**3**_**Sb**_**3**_**W**_**42**_**}** represent the first main group V heteropolyoxotungstates encapsulating a defect {(W^VI^O_7_)W^VI^_4_} (**{X**_**3**_**W**_**43**_**}**, X = Bi^III^ and Sb^III^) or full {(W^VI^O_7_)W^VI^_5_} (**{Co**_**3**_**Sb**_**3**_**W**_**42**_**}**) pentagonal unit. With 43 tungsten metal centers, **{X**_**3**_**W**_**43**_**}** (X = Bi^III^ and Sb^III^) are the largest unsubstituted tungstoantimonate– and bismuthate clusters reported to date. By using time-dependent UV/vis spectroscopy, the isostructural representatives **{Sb**_**3**_**W**_**43**_**}** and **{Bi**_**3**_**W**_**43**_**}** were subjected to a comprehensive study on their catalytic properties as homogeneous electron-transfer catalysts for the reduction of K_3_[Fe^III^(CN)_6_] as a model substrate revealing up to 5.8 times higher substrate conversions in the first 240 min (35% for **{Sb**_**3**_**W**_**43**_**}**, 29% for **{Bi**_**3**_**W**_**43**_**}**) as compared to the uncatalyzed reaction (<6% without catalyst after 240 min) under otherwise identical conditions.

## Abstract 2

We report on the synthesis and characterization of three new tungsten-based defect {(W^VI^O_7_)W^VI^_4_}K_*x*_Na_*y*_[H_2_(XW^VI^_9_O_33_)(W^VI^_5_O_12_)(X_2_W^VI^_29_O_103_)]·*n*H_2_O **{X**_**3**_**W**_**43**_**}** (*x* = 11, *y* = 16, and *n* = 115.5 for X = Sb^III^; *x* = 20, *y* = 7, and *n* = 68 for Bi^III^) or full pentagonal {(W^VI^O_7_)W^VI^_5_} unit K_8_Na_15_[H_16_(Co^II^(H_2_O)_2_)_0.9_(Co^II^(H_2_O)_3_)_2_(W^VI^_3.1_O_14_)(Sb^III^W^VI^_9_O_33_)(Sb^III^_2_W^VI^_30_O_106_)(H_2_O)]·53H_2_O **{Co**_**3**_**Sb**_**3**_**W**_**42**_**}** encapsulating main group V representatives. With 43 W centers, **{Sb**_**3**_**W**_**43**_**}** and **{Bi**_**3**_**W**_**43**_**}** exhibit the highest nuclearity among unsubstituted tungstoantimonates and bismuthates reported to date. The catalytic properties of **{Sb**_**3**_**W**_**43**_**}** and **{Bi**_**3**_**W**_**43**_**}** as homogeneous electron-transfer catalysts for the reduction of K_3_[Fe^III^(CN)_6_] to K_4_[Fe^II^(CN)_6_] was investigated.

## Introduction

Polyoxometalates (POMs)^1^ represent a broad class of anionic inorganic clusters with versatile structural topologies resulting in a variety of chemical and physical properties which can be modulated by molecular design. These features make them attractive materials in a wide range of fields, like catalysis,^2^ electrochemistry,^3^ magnetochemistry,^4^ and biological chemistry^5^ including protein crystallography,^6^ and the subject of challenging interdisciplinary research.^7^ In polyoxotungstate (POT) chemistry, architectural control can be achieved at the fragment level by carefully selecting the heteroanion template. Among the variety of primary heteroatoms grafted into heteropolyoxotungstates, main group V and VI representatives such as As^III^, Sb^III^, Bi^III^, Se^IV^, and Te^IV^ are of synthetic interest as their lone pair prevents the formation of a closed Keggin {XW_12_O_40_} sphere resulting in a structure-directing effect of the heteroanion. Using Se^IV^ and Te^IV^ as central atoms in anionic templates, a series of nanosized tungstoselenates and tellurates has been synthesized and characterized (Table S1). Similar to the molybdenum-based nanosized {Mo_154_} and {Mo_368_},^8^ the synthesized tungstoselenates and tellurates comprise full pentagonal {(W^VI^O_7_)W^VI^_5_} or defect pentagonal {(W^VI^O_7_)W^VI^_4_} units (Table S1), which are known as key building blocks in the formation of nanostructures.^9^ In contrast to their main group VI containing counterparts, the occurrence of full {(W^VI^O_7_)W^VI^_5_} or defect pentagonal {(W^VI^O_7_)W^VI^_4_} units has not been reported for main group V containing heteropolyoxotungstates yet (Table S1), although As^III^-, Sb^III^-, and Bi^III^-containing POTs like the Krebs archetype as their largest subclass have shown considerable importance in the field of both homo- and heterogeneous catalysis in the past.^2,10^ The first representatives of the Krebs archetype, [M_2_(H_2_O)_6_(WO_2_)_2_(β-SbW_9_O_33_)_2_]^(14–2*n*)–^ (M^*n*+^ = Fe^3+^, Co^2+^, Mn^2+^, and Ni^2+^), which are constituted by two lone-pair-containing β-Keggin lacunary fragments, e.g., [β-Sb^III^W_9_O_33_]^9–^, were reported by Bösing et al. in 1997.^11^ Since then, much attention has been paid to the catalytic properties of the Krebs archetype with recent focus on the use of tungstoantimonates as electron-transfer catalysts for the reductive conversion of pollutants such as K_3_[Fe^III^(CN)_6_] to K_4_[Fe^II^(CN)_6_].^12^ Herein, we report on the synthesis and characterization of K_20_Na_7_[H_2_(Bi^III^W^VI^_9_O_33_)(W_5_O_12_)(Bi^III^_2_W^VI^_29_O_103_)]·68H_2_O **{Bi**_**3**_**W**_**43**_**}**, K_11_Na_16_[H_2_(Sb^III^W^VI^_9_O_33_)(W_5_O_12_)(Sb^III^_2_W^VI^_29_O_103_)]·115.5H_2_O **{Sb**_**3**_**W**_**43**_**}**, and K_8_Na_15_[H_16_(Co^II^(H_2_O)_2_)_0.9_(Co^II^(H_2_O)_3_)_2_(W^VI^_3.1_O_14_)(Sb^III^W^VI^_9_O_33_)(Sb^III^_2_W^VI^_30_O_106_)(H_2_O)]·53H_2_O **{Co**_**3**_**Sb**_**3**_**W**_**42**_**}**, which were synthesized using a one-pot procedure and a stepwise approach, respectively. Being the first main group V heteropolyoxotungstates that incorporate a defect pentagonal {(W^VI^O_7_)W^VI^_4_} unit, **{Bi**_**3**_**W**_**43**_**}** and **{Sb**_**3**_**W**_**43**_**}** represent the largest unsubstituted tungstobismuthates and -antimonates reported so far (Table S2). The isostructural representatives **{Sb**_**3**_**W**_**43**_**}** and **{Bi**_**3**_**W**_**43**_**}** were subjected to a comprehensive study on their performance as electron-transfer catalysts for the reductive conversion of K_3_[Fe^III^(CN)_6_] and their stability under turnover conditions was confirmed by recyclability experiments accompanied by postcatalytic IR spectroscopic studies.

## Results and Discussion

### Synthesis

**Scheme 1:**
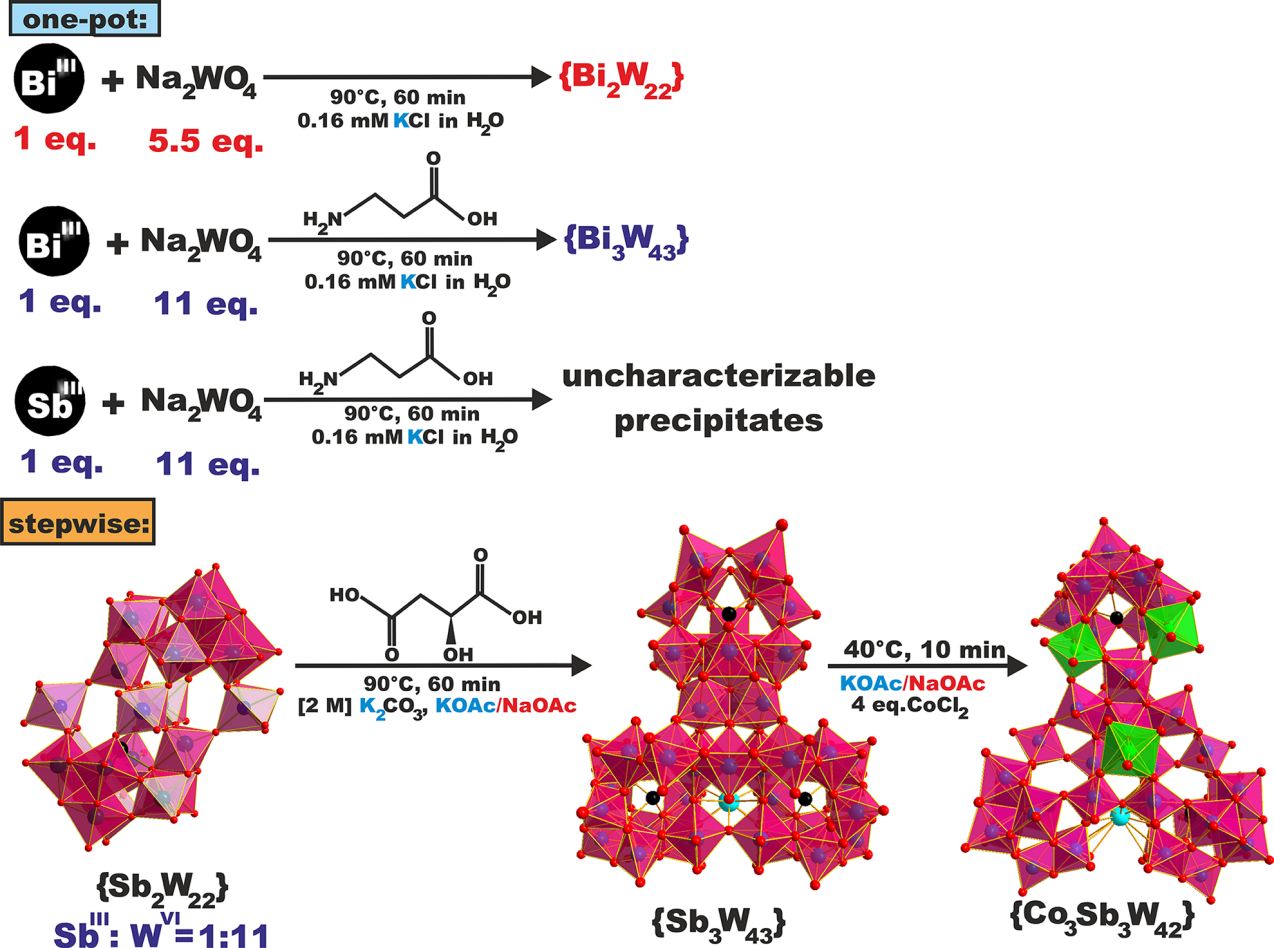
Schematic Representation Showing the Synthesis of **{X**_**3**_**W**_**43**_**}** (X = Sb^III^ and Bi^III^) and **{Co**_**3**_**Sb**_**3**_**W**_**42**_**}** Taking the elevated potassium and acetate content as well as the use of a carboxylic acid and a X/W = 1:11 ratio as key parameters for the one-pot synthesis of **{Bi**_**3**_**W**_**43**_**}** into account, the step-by-step protocol for the synthesis of **{Sb**_**3**_**W**_**43**_**}** was designed. By using the nonlacunary **{Sb**_**2**_**W**_**22**_**}** Krebs archetype, which was prepared in a first step as a precursor together with l-malic acid in a KOAc/NaOAc (5% *v*/*v*) mixture, **{Sb**_**3**_**W**_**43**_**}** is obtained. Addition of **{Sb**_**3**_**W**_**43**_**}** to a CoCl_2_ solution in KOAc/NaOAc (5% *v*/*v*) leads to the formation of **{Co**_**3**_**Sb**_**3**_**W**_**42**_**}** in a consecutive reaction step. Black, turquoise, and red spheres represent the X = Sb^III^ and Bi^III^, K^+^, and oxygen ions, respectively. Magenta and green transparent polyhedra represent {W^VI^O_6_} and {Co^II^O_6_}.

The new **{Bi**_**3**_**W**_**43**_**}** is prepared by a one-pot approach (Scheme 1). Given the well-documented directing effect of counter cations such as potassium for the formation of nanoclusters^13,14b^ along with the role of acetate in preventing the formation of the classical {W_11_} isopolyoxotungstate fragment,^15^ Bi(OAc)_3_ was chosen as a lone-pair-containing salt, and the reaction was carried out in a [0.16 mM] KCl solution with a W^VI^/Bi^III^ ratio of 11. Addition of β-alanine as a carboxylic acid source to the reaction mixture, which was heated to 90 °C for 1 h (Scheme 1) and consecutively filtered, resulted in the formation of needle-shaped single crystals of **{Bi**_**3**_**W**_**43**_**}** after 4 days. Despite all our efforts, single crystals of **{Bi**_**3**_**W**_**43**_**}** with sufficient quality for single-crystal XRD measurements could not be obtained. However, elemental analysis via ICP-MS, capillary ion electrophoresis (CIE) and EDX analysis (Figures S4–S6), IR spectroscopy (Figures S1–S3, Table S3), thermogravimetric analysis (TGA) (Figure S11, Table S5), and powder XRD measurements (Figure S14) clearly indicate the successful synthesis of pure **{Bi**_**3**_**W**_**43**_**}**. Importantly, decreasing the W/Bi ratio, e.g., from 11 to 5.5, by elevating the Bi(OAc)_3_ amount to increase the yield, resulted in exclusive formation of Krebs-type Na_12_[Bi_2_W_22_O_74_(OH)_2_]·44H_2_O^16^ highlighting the importance of a high W/X ratio as a key factor to prevent formation of Krebs POT, which is in accordance with the findings reported by Cronin and co-workers.^17^ To explore the role of β-alanine in the reaction system, various reaction conditions including systems with other carboxylic acids such as l-malic acid or α-alanine as well as systems lacking β-alanine at all were tested. In all cases, either uncharacterizable, inhomogeneous precipitates or single crystals of Krebs-type Na_12_[Bi_2_W_22_O_74_(OH)_2_]·44H_2_O were obtained, thereby highlighting the crucial role of β-alanine for the formation of **{Bi**_**3**_**W**_**43**_**}** in the reaction system. Attempts to prepare the antimony-containing counterpart **{Sb**_**3**_**W**_**43**_**}** via a one-pot approach by exchanging the lone-pair heteroanion source were unsuccessful and exclusively resulted in precipitates which could not be characterized.

Hence, a step-by-step protocol based on the key parameters determined for the one-pot synthesis of **{Bi**_**3**_**W**_**43**_**}** using the Krebs archetype K_12_[Sb_2_W_22_O_74_(OH)_2_]·27H_2_O **{Sb**_**2**_**W**_**22**_**}**(11) as a nonlacunary precursor^14^ was developed for the synthesis of **{Sb**_**3**_**W**_**43**_**}**. The synthesis of **{Sb**_**3**_**W**_**43**_**}** starts with the preparation of the literature-known nonlacunary Krebs-type precursor **{Sb**_**2**_**W**_**22**_**}**,^11^ which was chosen based on its W^VI^/Sb^III^ ratio of 11 (Scheme 1). Considering the crucial role of acetate and potassium ions in templating the formation of **{Bi**_**3**_**W**_**43**_**}**, an acetate buffer with slightly increased potassium content in the reaction mixture was applied to favor the formation and crystallization of the desired **{Sb**_**3**_**W**_**43**_**}**. Initial attempts to increase the potassium content of the reaction mixture by merely adding KCl resulted in precipitates, which despite all our efforts could not be characterized. The appearance of insoluble precipitates upon addition of KCl to the reaction mixture could be explained by a change of the ionic strength in the solution. Thus, the potassium content in the reaction mixture was elevated by dropwise addition of 2 M K_2_CO_3_ to a solution of **{Sb**_**2**_**W**_**22**_**}** in a KOAc/NaOAc (5% *v*/*v*) mixture. Consequently, 2 equivalents of l-malic acid as a carboxylic acid source with respect to **{Sb**_**2**_**W**_**22**_**}** were added to the solution, and the resulting colorless reaction mixture was heated to 90 °C for 60 min. Slow evaporation of the reaction mixture at 20 °C resulted in rhombohedral crystals of **{Sb**_**3**_**W**_**43**_**}** (CCDC 2070328) in a 20% yield based on tungsten after 4 days (Scheme 1). To explore the role of l-malic acid, synthesis studies lacking the carboxylic acid were conducted, resulting in a Krebs-POT K_12_[Sb_2_W_22_O_74_(OH)_2_]·27H_2_O (CCDC 406487).^11^ It should be mentioned that a one-pot synthesis approach by mixing WO_4_^2–^, Sb^3+^, and l-malic acid in the corresponding stoichiometric ratios exclusively resulted in the formation of Krebs POM or uncharacterizable, inhomogeneous precipitates, respectively. **{Co**_**3**_**Sb**_**3**_**W**_**42**_**}** was prepared in a consecutive reaction step by addition of **{Sb**_**3**_**W**_**43**_**}** to a solution of CoCl_2_ (4 eq. with respect to **{Sb**_**3**_**W**_**43**_**}**) in a KOAc/NaOAc (5% *v*/*v*) mixture. Mild heating of the pink reaction mixture to 40 °C for 10 min yielded complete dissolution of **{Sb**_**3**_**W**_**43**_**}**, and subsequent filtration resulted in the formation of pink plate-shaped crystals of **{Co**_**3**_**Sb**_**3**_**W**_**42**_**}** after 4 days (CCDC 2070860) (Scheme 1). It should be mentioned that a one-pot approach to prepare **{Co**_**3**_**Sb**_**3**_**W**_**42**_**}** by mixing CoCl_2_, Na_2_WO_4_, and Sb_2_O_3_ or Sb(OAc)_3_ in the corresponding stoichiometric ratios exclusively resulted in the formation of a Co^II^ disubstituted Krebs POT,^11,16^ [Sb_2_W_20_Co_2_O_70_(H_2_O)_6_]^10-^, as confirmed by SXRD studies.

### Structure

**Figure 1:**
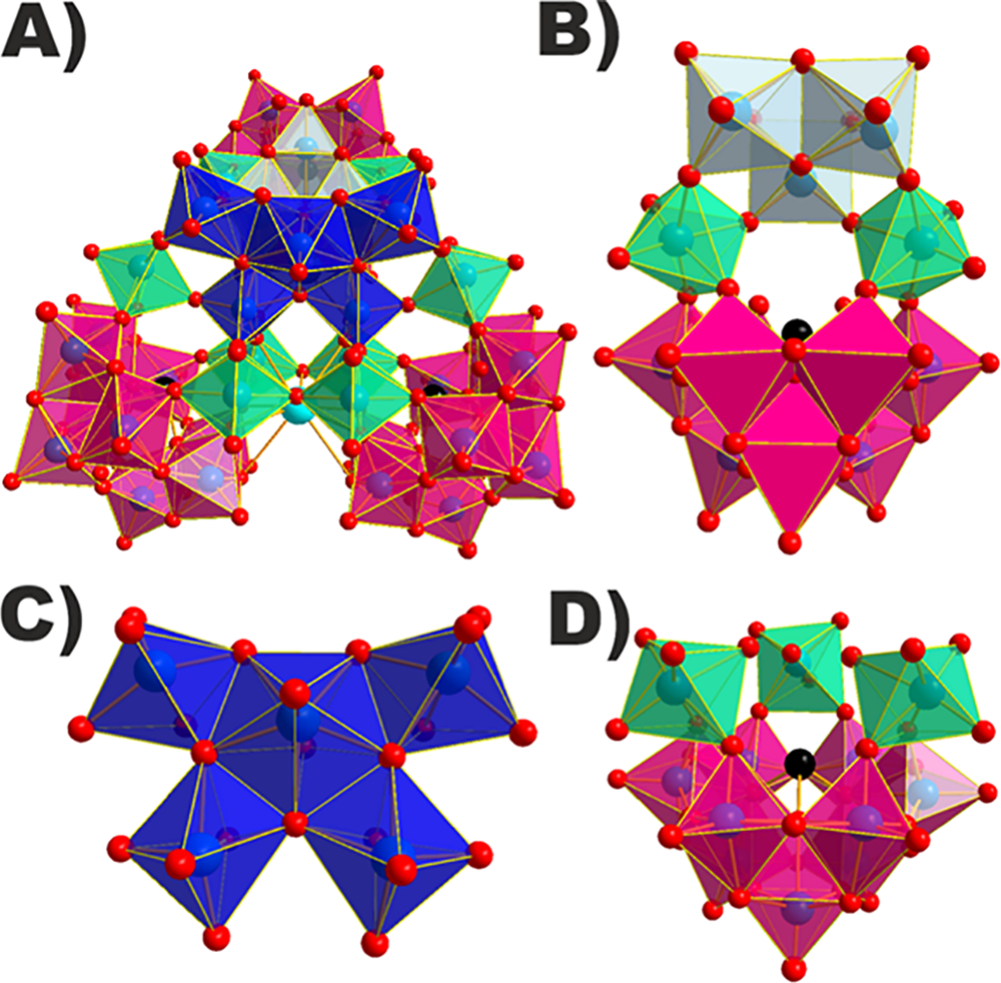
Polyhedral representation of **{X**_**3**_**W**_**43**_**}** (X = Sb^III^ and Bi^III^) showing the polyanion in a frontal view (A) as well as the central {W_3_O_13_} moiety (B) which is connected to a defect pentagonal {(W^VI^O_7_)W^VI^_4_} unit (C). Six single {WO_6_} linkers (D) complete the structure by connecting the {(W^VI^O_7_)W^VI^_4_} unit to {XW_9_} building blocks. Black and red spheres represent the X = Sb^III^ and Bi^III^ and oxygen ions, respectively. Magenta, ice gray, turquoise, and royal blue transparent polyhedra represent the {XW_9_} building blocks, the {W_3_O_13_} unit, single {WO_6_} linkers, and {(W^VI^O_7_)W^VI^_4_} unit, respectively.

**Figure 2:**
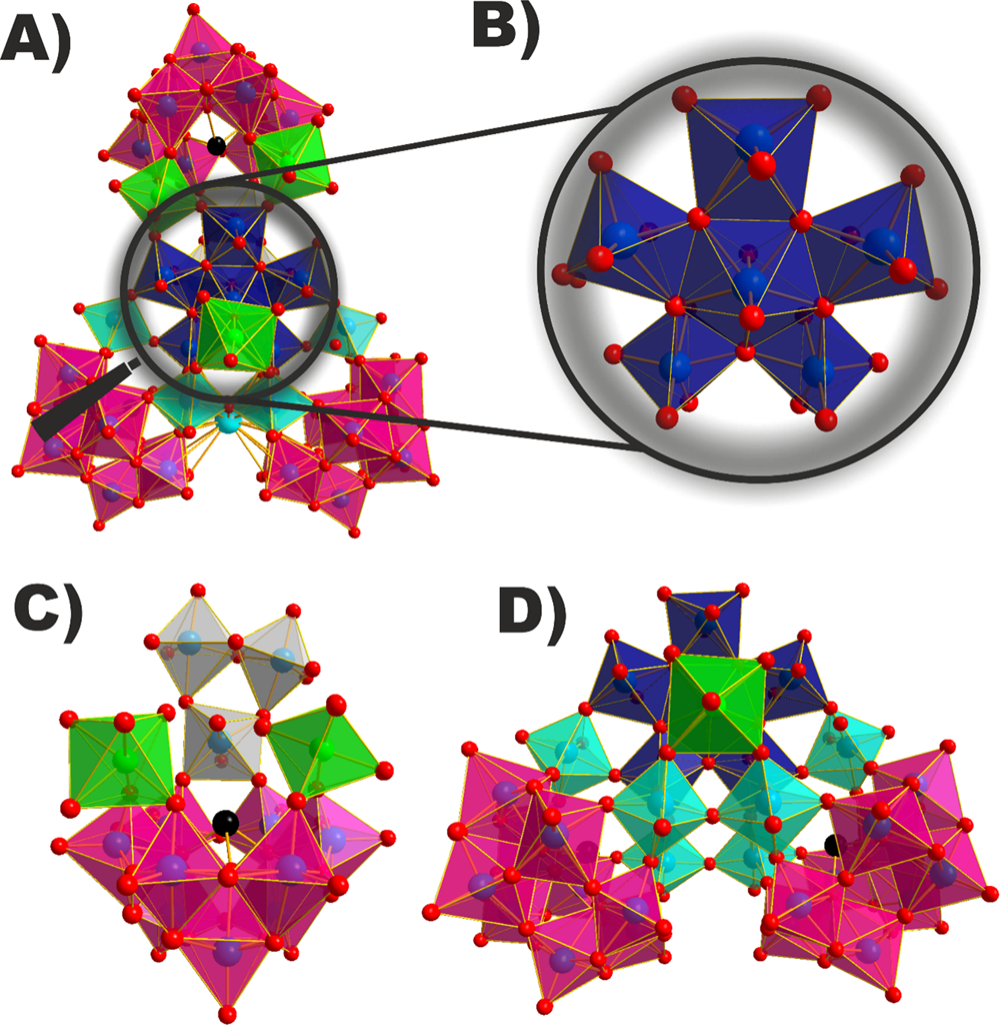
Polyhedral representation of **{Co**_**3**_**Sb**_**3**_**W**_**42**_**}** showing the polyanion in a frontal view (A) as well as the full pentagonal {(W^VI^O_7_)W^VI^_5_} unit (B) which is connected to the {Sb^III^W_9_} moiety via two {Co^II^O_6_} linkers and a {W_3_O_14_} unit (C). A third {Co^II^O_6_} octahedron present in the {Sb_2_W_30_} unit completes the structure (D). Black, turquoise, and red spheres represent the Sb^III^, K^+^, and oxygen ions, respectively. Magenta, ice gray, turquoise, green, and royal blue transparent polyhedra represent the {XW_9_} building blocks, the {W_3_O_14_} unit, single {WO_6_} linkers, {Co^II^O_6_} octahedra, and {(W^VI^O_7_)W^VI^_5_} unit, respectively.

Single-crystal X-ray diffraction (SXRD) measurements were performed revealing that **{Sb**_**3**_**W**_**43**_**}** (Tables S7–S9) and **{Co**_**3**_**Sb**_**3**_**W**_**42**_**}** (Tables S7, S10, and S11) crystallize in the triclinic space group *P*1̅. The architecture of **{Sb**_**3**_**W**_**43**_**}** presents three **{SbW**_**9**_**}** subunits with an average Sb–O bond length of 1.99(1) Å and a metal core composed of 16 W centers. The {W_16_} core contains the rarely reported {W^VI^O_7_} building block comprising a defect pentagonal {(W^VI^O_7_)W^VI^_4_} unit (Figure 1C) together with two corner-sharing {WO_6_} (Figure 1D), as well as a doubly protonated {W_3_O_13_} unit (Figure 1B) and six {WO_6_} linkers (Figure 1B,D). Three **{SbW**_**9**_**}** units encapsulating the {W_16_} core complete the overall cluster (Figure 1A). The average W–O bond length in the pentagonal {WO_7_} unit is 2.01(3) Å which is slightly longer than that determined in the {WO_6_} units. **{Co**_**3**_**Sb**_**3**_**W**_**42**_**}** exhibits a compositional similarity to its precursor **{Sb**_**3**_**W**_**43**_**}** as both polyanions have two main parts, that is, one {Sb_2_W_29_} type subunit (for **{Co**_**3**_**Sb**_**3**_**W**_**42**_**}** it is {Sb_2_W_30_}, Figure 2D) and one ***B*****-β-{SbW**_**9**_**}** unit (Figure 2C). In **{Co**_**3**_**Sb**_**3**_**W**_**42**_**}**, these two parts are connected by three kinds of linkers: one {W_3_O_14_} unit and two {CoO_6_} octahedra (Figure 2C). A third {CoO_6_} octahedron is located at the {Sb_2_W_30_} unit which has the same structure as the {Sb_2_W_29_} moiety in **{Sb**_**3**_**W**_**43**_**}** with the lacunary pentagonal unit {(W^VI^O_7_)W^VI^_4_} being a completed {(W^VI^O_7_)W^VI^_5_} (Figure 2A,B) core. The average W–O bond length within the pentagon-shaped {WO_7_} unit is 2.00(9) Å and thereby slightly shorter than that present in anion **{Sb**_**3**_**W**_**43**_**}**. The oxidation state of the cobalt centers in **{Co**_**3**_**Sb**_**3**_**W**_**42**_**}** was investigated by X-ray photoelectron spectroscopy (XPS) (Figures S7–S9). Using a Gaussian fitting method, the Co 2p core levels were fit, giving rise to Co 2p_3/2_ and Co 2p_1/2_ peaks at binding energies of 796.98 and 781.38 eV and two shake-up satellites (Figure S9), respectively, which are characteristic of Co^2+^ centers as suggested by BVS calculations.^18^

The numbers of water molecules in **{Sb**_**3**_**W**_**43**_**}**·115.5H_2_O, **{Bi**_**3**_**W**_**43**_**}**·68H_2_O, and **{Co**_**3**_**Sb**_**3**_**W**_**42**_**}**·62H_2_O were determined using thermogravimetric analysis (TGA). The four weight-loss regions observed for the isostructural **{Sb**_**3**_**W**_**43**_**}** and **{Bi**_**3**_**W**_**43**_**}** (Figure S10 and S11, Tables S4 and S5) are attributed to losses of 115.5 and 68 water molecules, respectively, whereas **{Co**_**3**_**Sb**_**3**_**W**_**42**_**}** exhibits three weight-loss steps attributed to the loss of 62 water molecules (Figure S12, Table S6). Powder XRD measurements were performed on the three polyanions **{Sb**_**3**_**W**_**43**_**}**, **{Bi**_**3**_**W**_**43**_**}**, and **{Co**_**3**_**Sb**_**3**_**W**_**42**_**}** and compared to the corresponding simulated diffractogram (Figures S13–S15). The powder diagrams of the bulk samples indicated the inhomogeneity caused by gradual loss of crystal water as shown by the compounds’ TGA curves, which revealed water loss beginning at room temperature (298 K) (Figures S10–S12), commonly observed for high-nuclear polyoxometalates.^17^ Apart from XRD, all three POTs were characterized in the solid state by ATR-IR spectroscopy showing the terminal W=O and bridging W–O–W vibrations typical for the Keggin-type polyoxotungstate framework (Figures S1–S3, Table S3). The UV/vis spectra of **{X**_**3**_**W**_**43**_**}** and **{Co**_**3**_**Sb**_**3**_**W**_**42**_**}** are characterized by an absorption maximum at ∼205 nm with a shoulder at ∼250 nm attributed to the p_π_(O_b_) → d_π_*(W) ligand-to-metal charge-transfer (LMCT) transitions typical for the Keggin-type framework (Figure S16).^19^ In addition to the LMCT transitions associated with its POT framework, the visible spectrum of **{Co**_**3**_**Sb**_**3**_**W**_**42**_**}** displays a peak located at ∼548 nm, which is typical for octahedrally coordinated Co(II) metal centers (Figure S17).^20^

### Catalytic Studies

**Figure 3:**
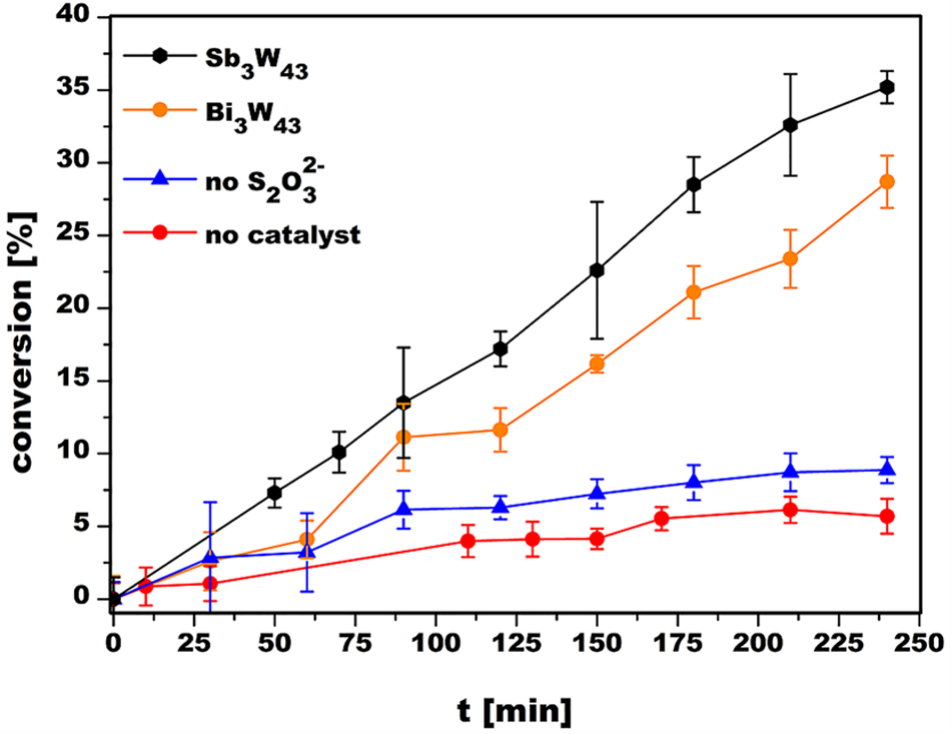
Time-dependent conversion of K_3_[Fe^III^(CN)_6_] to K_3_[Fe^II^(CN)_6_] catalyzed by **{Bi**_**3**_**W**_**43**_**}** and **{Sb**_**3**_**W**_**43**_**}**. Control experiments lacking catalyst or S_2_O_3_^2–^ show a significant drop in conversion (<6%) under otherwise identical conditions.

Potassium ferricyanide (K_3_[Fe^III^(CN)_6_]) is known to be one of the most common contaminations in polluted water, air, and soil.^21^ Considering the acute toxicity, mutagenicity, carcinogenicity, and easy accumulation in the human body, aquatic animals, and various living organisms, the reductive decontamination of K_3_[Fe^III^(CN)_6_] into nontoxic K_4_[Fe^II^(CN)_6_]^22^ is generally achieved by using noble metals as catalysts, such as Au^23^ or Ru.^24^ However, the high cost and low abundance of noble metals limits their widespread application rendering polyoxometalates with their promising redox properties and reversible electron gain-and-loss capacities to be interesting cost-effective electron-transfer catalysts (Scheme S1).^12,18,25^ In this work, the catalytic reduction reaction of K_3_[Fe^III^(CN)_6_] to K_4_[Fe^II^(CN)_6_] using new isostructural **{Bi**_**3**_**W**_**43**_**}** and **{Sb**_**3**_**W**_**43**_**}** as homogeneous electron-transfer catalysts was investigated. Considering the characteristic absorption band that K_3_[Fe^III^(CN)_6_] displays at 420 nm attributed to a ^2^T_1g_ → ^2^T_2g_ transition in aqueous solution^26^ (Figure S18), the stepwise conversion of the model substrate [Fe^III^(CN)_6_]^3–^ could be followed using time-dependent UV/vis spectroscopy by measuring a standard curve of [Fe^III^(CN)_6_]^3–^ to ensure the reliability of the experimental setup (Figures S19 and S20). To probe the catalytic properties of **{Bi**_**3**_**W**_**43**_**}** and **{Sb**_**3**_**W**_**43**_**}**, 1.5 mL of a stock solution of 80 μM catalyst, 1 mM K_3_[Fe^III^(CN)_6_], and 8.7 mM Na_2_S_2_O_3_ as a reducing agent was heated at 55 °C in water (pH 6.8 via HCl [1 M]), and aliquots of the reaction mixture after 0, 30, 60, 90, 120, 150, 180, 210, and 240 min were taken and subjected to UV/vis spectroscopy (Figures 3 and S21C,D). For all reactions, the concentration of Na_2_S_2_O_3_ was chosen to exceed the concentration of K_3_[Fe^III^(CN)_6_] to ensure a pseudo-first-order reaction for the reduction process (Figures S22 and S23). The turnover frequency values (TOF) were calculated based on the substrate conversion determined from the absorption at 420 nm after 180 min. The TOF is 1.19 h^–1^ for **{Sb**_**3**_**W**_**43**_**}**, which is comparable to other previously reported tungstoantimonates such as (H_2_en)_7_[(WO_2_)_2_(WO_3_)_2_(*B*-β-SbW_9_O_33_)_2_]·12H_2_O (en = ethylenediamine) (TOF = 1.64 h^–1^) under similar reaction conditions,^12^ while TOF is 0.89 h^–1^ for **{Bi**_**3**_**W**_**43**_**}**. The lower catalytic performance of **{Bi**_**3**_**W**_**43**_**}** compared to that of **{Sb**_**3**_**W**_**43**_**}** can be explained by the change of the primary heteroatom in the otherwise isostructural polyanion leading to a change of the overall redox properties of the polyanion, which has been previously observed for other POM-based catalysts.^27^ Considering the instability of **{Co**_**3**_**Sb**_**3**_**W**_**42**_**}** under turnover conditions indicated by leeching of free Co(II) species (Figure S24), an increase in the absorption at ∼420 nm could be observed rendering a reliable interpretation of the UV/vis data difficult. Control experiments lacking the catalyst (Figure S21A) or Na_2_S_2_O_3_ (Figure S21B) under otherwise identical conditions resulted in similarly negligible conversion of the substrate (∼6% after 240 min at 55 °C), which indicates that the presence of Na_2_S_2_O_3_ as a reducing agent is crucial for the conversion to take place and highlights the catalytic role of **{Sb**_**3**_**W**_**43**_**}** and **{Bi**_**3**_**W**_**43**_**}** for the electron transfer (Figures 3 and S25).

### Postcatalytic Studies

Solution stability studies on **{Sb**_**3**_**W**_**43**_**}** and **{Bi**_**3**_**W**_**43**_**}** after reaction with K_3_[Fe^III^(CN)_6_] were intended to be performed; however, due to the low solubility and sensitivity of the ^183^W nucleus (14.3% natural abundance), the ^183^W NMR measurements at conditions pertinent to the catalytic reactions were not informative, which is a common problem encountered in postcatalytic POM characterization.^28^ Considering the low solubility of **{Sb**_**3**_**W**_**43**_**}** and **{Bi**_**3**_**W**_**43**_**}**, the POM concentrations used for the catalytic studies were too low to obtain reasonable amounts for consecutive post catalytic PXRD measurements. Hence, to investigate the postcatalytic stability of **{Sb**_**3**_**W**_**43**_**}** and **{Bi**_**3**_**W**_**43**_**}** indirectly, the IR spectra of the polyanions were recorded after precipitation with cesium chloride, thereby clearly showing the characteristic W–O–W bridging and terminal W=O vibration bands in the tungsten fingerprint area from 300–1000 cm^–1^, which indicates the solution stability of the polyanions after reaction with K_3_[Fe^III^(CN)_6_] (Figures S26 and S27), and represents an established method generally used for the postcatalytic study of POMs.^27,29^ Moreover, the time-dependent UV/vis spectra of the reaction mixture after POM precipitation and the subsequent addition of 1 mM K_3_[Fe^III^(CN)_6_] substrate to initiate a second reaction cycle resembled that of a blank experiment lacking the catalyst (∼8% conversion after 170 min, Figures S28 and S29), thereby suggesting complete removal of the corresponding POT catalysts upon addition of cesium chloride.

The recyclability of **{Sb**_**3**_**W**_**43**_**}** and **{Bi**_**3**_**W**_**43**_**}** as electron-transfer catalysts was tested in a consecutive experiment by reloading the reaction mixture with K_3_[Fe^III^(CN)_6_] substrate and reducing agent Na_2_S_2_O_3_ after UV/vis measurements confirmed the complete conversion (98%) of K_3_[Fe^III^(CN)_6_] (Figures S30 and S31). A direct comparison of the TOF values obtained in the first (TOF_cycle1_ = 0.89 h^–1^ for **{Bi**_**3**_**W**_**43**_**}**, TOF_cycle1_ = 1.19 h^–1^ for **{Sb**_**3**_**W**_**43**_**}**) and the second (TOF_cycle2_ = 1.04 h^–1^ for **{Bi**_**3**_**W**_**43**_**}**, TOF_cycle2_ = 1.85 h^–1^ for **{Sb**_**3**_**W**_**43**_**}**) reaction cycles indicates the slightly increased TOFs for the second reaction cycles. Considering the incomplete consumption of S_2_O_3_^2–^ during the first reaction cycle, the additional reducing agent resulted in overall higher amounts of S_2_O_3_^2–^ in the second cycle, thereby eventually leading to an increased catalytic performance of the system and thus higher TOF values. This assumption is supported by control experiments using varying concentrations of S_2_O_3_^2–^ revealing an increase in catalytic performance with higher S_2_O_3_^2–^ amounts (Figure S25), thus demonstrating the recyclability of **{Sb**_**3**_**W**_**43**_**}** and **{Bi**_**3**_**W**_**43**_**}** as electron-transfer catalysts.

## Conclusions

In conclusion, the first main group V heteropolytungstates encapsulating the rarely reported tungsten-based pentagonal unit have been synthesized and characterized using one-pot and step-by-step procedures. The key factors that led to the one-pot assembly of **{Bi**_**3**_**W**_**43**_**}**, such as elevated potassium and acetate contents as well as the presence of a carboxylic acid, were subsequently used to develop a rational step-by-step protocol applying the Krebs archetype as a nonlacunary precursor to yield the antimony containing counterpart **{Sb**_**3**_**W**_**43**_**}** and subsequently cobalt-trisubstituted product **{Co**_**3**_**Sb**_**3**_**W**_**42**_**}**. Isostructural representatives **{Bi**_**3**_**W**_**43**_**}** and **{Sb**_**3**_**W**_**43**_**}** were shown to be highly active, stable, and recyclable homogeneous electron-transfer catalysts for the reductive conversion of K_3_[Fe^III^(CN)_6_] to K_4_[Fe^II^(CN)_6_] and a direct comparison of their catalytic performances revealed higher catalytic activity for **{Sb**_**3**_**W**_**43**_**}** as compared to **{Bi**_**3**_**W**_**43**_**}** thereby suggesting the primary heteroatom Sb^III^ to have a modulating effect on the overall redox properties of the otherwise isostructural polyanions. This work promotes the use of nonlacunary lone-pair containing POTs as precursors to expand the family of pentagonal unit encapsulating nanosized POTs with catalytically promising properties which can be tuned by changing the primary heteroatom thereby paving the way for cost-effective electron-transfer catalysts.

## Supporting Information Available

Details on synthesis, IR, TGA, XRD, EDX, XPS, and UV/vis spectroscopy (PDF)

## Accession Codes

CCDC 2070328 and 2070860 contain the supplementary crystallographic data for this paper. These data can be obtained free of charge via www.ccdc.cam.ac.uk/data_request/cif, or by emailing data_request@ccdc.cam.ac.uk, or by contacting The Cambridge Crystallographic Data Centre, 12 Union Road, Cambridge CB2 1EZ, UK; fax: +44 1223 336033.
