# Supplementary material for: Defect {(WVIO7)WVI4} and Full {(WVIO7)WVI5} Pentagonal Units as Synthons for the Generation of Nanosized Main Group V Heteropolyoxotungstates
Source: Inorg Chem. 2021 Jun 4;60(12):8917–23. doi: 10.1021/acs.inorgchem.1c00810 (PMC8220499; doi:10.1021/acs.inorgchem.1c00810)
Supplement: Supplementary file 1 — ic1c00810_si_001.pdf [file ic1c00810_si_001.pdf]

ELECTRONIC SUPPLEMENTARY INFORMATION

Defect $\{(W^{VI}O_7)W^{VI}_4\}$ – and full $\{(W^{VI}O_7)W^{VI}_5\}$ pentagonal units as synthons for the generation of nanosized main group V heteropolyoxotungstates

Elias Tanuhadi[†], Nadiia I. Gumerova[†], Alexander Prado-Roller[‡], Andreas Mautner[⊥] and Annette Rompel[*, †]

[*]correspondence to annette.rompel@univie.ac.at

[†] Universität Wien, Fakultät für Chemie, Institut für Biophysikalische Chemie, Althanstr. 14, 1090 Wien, Austria. www.bpc.univie.ac.at

[‡] Universität Wien, Fakultät für Chemie, Zentrum für Röntgenstrukturanalyse und Institut für Anorganische Chemie, Währinger Str. 42, 1090 Wien, Austria

[⊥] Universität Wien, Fakultät für Chemie, Polymer and Composite Engineering (PaCE) Group, Institute of Materials Chemistry and Research, Währinger Str. 42, 1090 Vienna, Austria

Content

1. **General Information** ... 3
2. **Experimental Procedure** ... 4
   2.1. Preparation of $K_{11}Na_{16}[H_2(SbW_9O_{33})(W_5O_{12})(Sb_2W_{29}O_{103})]$ • 115.5 H_2O **{Sb_3W_{43}}** ... 4
   2.2. Preparation of $K_8Na_{15}[H_{16}(Co(H_2O)_2)_{0.9}(Co(H_2O)_3)_2(W_{3.1}O_{14})(SbW_9O_{33})(Sb_2W_{30}O_{106})(H_2O)]$ • 53 H_2O **{$Co_3Sb_3W_{42}$}** ... 4
   2.3. Preparation of $K_{20}Na_7[H_2(BiW_9O_{33})(W_5O_{12})(Bi_2W_{29}O_{103})]$ • 68 H_2O **{Bi_3W_{43}}** ... 4
3. **IR-spectra** ... 7
4. **Thermogravimetric Analysis** ... 15
5. **Single-Crystal X-ray Diffraction** ... 18
6. **Powder X-ray Diffraction** ... 20
7. **UV/Vis Spectroscopy** ... 23
8. **Catalysis** ... 25
9. **Post-Catalysis** ... 33
   9.1. Post-catalytic POM-precipitation for subsequent analysis with ATR-IR ... 33
   9.2. Reloading experiment of the reaction mixture after post-catalytic POM-precipitation ... 35
   9.3. Recyclability of **{Sb_3W_{43}}** and **{Bi_3W_{43}}** ... 37
10. **References** ... 40

1. General Information

All reagents and chemicals were of high-purity grade and were used as purchased without further purification. $Na_9[B\text{-}\alpha\text{-}SbW_9O_{33}]$ and $K_{12}[Sb_2W_{22}O_{74}(OH)_2] \cdot 27\ H_2O$ **{Sb_2W_{22}}** were prepared according to the literature procedure.[1] The 2:1 KOAc/NaOAc 5% (v/v) pH 5.5 acetate buffer [2 M] was prepared by mixing 5 ml of a KOAc/AcOH buffer (pH 5.5, [2 M]) with 95 ml of a NaOAc/AcOH buffer solution (pH 5.5, [2 M]).

Elemental analysis: Elemental analysis of W, Bi and Sb contents was performed using a Perkin Elmer Elan 6000 ICP-MS in aqueous solutions containing 2 % ultrapure HNO_3 (**{Sb_3W_{43}}, {Bi_3W_{43}}**) and a Thermo Scientific Nexsa Photoelectron Spectrometer using Al *Kα* X-rays as source (X-ray photoelectron spectroscopy XPS) (**{$Co_3Sb_3W_{42}$}**). XPS was performed on a spot size of 400 μm with an energy step size of 1 eV for the survey and 0.1 eV for detailed analysis, respectively. Homogenized powdered samples were etched for 60 s using a low-energy Ar cluster (6000 eV, 1000 atom clusters) prior to analysis to clean the surface of the sample. To assess the reliability of the results obtained from XPS, control measurements were performed on powdered samples of the literature known POT compound $K_6[P_2W_{18}O_{62}] \cdot 14\ H_2O$[2] used as a benchmark that was characterized in solution and in the solid state. The XPS measurements on the benchmark compound gave reliable results (Anal. Calcd. (%) for $K_6P_2W_{18}O_{76}H_{28}$): K, 4.84; P, 1.28; W, 68.23; Found: K, 6.53; P, 1.9; W, 68.59. The K and Na contents were determined using a Perkin Elmer 1100 Flame AAS (**{Sb_3W_{43}}**), a CRYSTAL 310 Capillary Electrophoresis equipped with a CRYSTAL 1.000 Conductivity Detector (ATI–Unicam, 1995) **{Bi_3W_{43}}** and a Thermo Scientific Nexsa Photoelectron Spectrometer using Al *Kα* X-rays as source (X-ray photoelectron spectroscopy XPS) (**{$Co_3Sb_3W_{42}$}**), respectively.

Energy dispersive X-ray (EDX) analysis data were obtained using an Oxford Instruments INCA Energy SEM-EDS system.

Attenuated total reflection Fourier−transform Infrared Spectroscopy: All spectra were recorded on a Bruker Vertex70 IR Spectrometer equipped with a single−reflection diamond−ATR unit. Frequencies are given in cm^{-1}, intensities denoted as w = weak, m = medium, s = strong.

Thermogravimetric analysis (TGA): was performed on a Mettler SDTA851e Thermogravimetric Analyzer under N_2 flow with a heating rate of 5 K min^{-1} in the region 298−973 K.

Single crystal X-ray diffraction (SXRD): The X-ray data were measured on a Bruker D8 Venture equipped with a multilayer monochromator, *MoK/α* (λ = 0.71073Å) (for **{Sb_3W_{43}}**) INCOATEC micro focus sealed tube or *CuK/α* (λ = 1.54178) (for **{$Co_3Sb_3W_{42}$}**), and Oxford cooling device. The structures were solved by direct methods and refined by full-matrix least-squares. Non-hydrogen atoms were refined with anisotropic displacement parameters. The following software was used for the structure-solving procedure: frame integration, Bruker SAINT software package using a narrow-frame algorithm (absorption correction)[3], SADABS[4], SHELXS-2013[5] (structure solution), SHELXL-2013[6] (refinement), OLEX2[7] (structure solution, refinement, molecular diagrams, and graphical user-interface), and SHELXLE[8] (molecular diagrams and graphical user interface). Experimental data and CCDC-codes are provided in **Tables S7-S11**. Due to the weak diffracting nature of **{$Co_3Sb_3W_{42}$}**, despite using Cu radiation and careful selection of crystals, data had to be truncated to include only the portion in which the strongest reflections were observed.

Powder X-ray diffraction was performed on an EMPYREAN diffractometer system using Cu Kα radiation (λ = 1.540598), a PIXcel3D-Medipix3 1 × 1 detector (used as a scanning line detector) and a divergence slit fixed at 0.1 mm. The scan range was from 8° to 50° (2θ).

UV/Vis spectroscopy: UV/Vis spectra were collected on a Shimadzu UV 1800 spectrophotometer.

2. Experimental Procedure

2.1. Preparation of $K_{11}Na_{16}[H_2(SbW_9O_{33})(W_5O_{12})(Sb_2W_{29}O_{103})]$ • 115.5 H_2O {Sb_3W_{43}}

To a stirred solution of **{Sb_2W_{22}}** (300 mg, 0.051 mmol) in a mixture of 10 ml KOAc/NaOAc 5% (v/v) pH 5.5 acetate buffer and 5 ml H_2O at 90°C, L-malic acid (13.4 mg, 0.034 mmol, 2 eq. with respect to **{Sb_2W_{22}}**) was added followed by dropwise addition of 60 µL of a [2 M] K_2CO_3 solution. After stirring for 1 h at 90°C, the reaction mixture was left to evaporate at room temperature giving colorless block shaped crystals of **{Sb_3W_{43}}** after approx. 2-3 days. Yield: 137 mg, 20% based on W. Anal. Calcd. (%) for $K_{11}Na_{16}H_{233}Sb_3W_{43}O_{263}$ ($K_{11}Na_{16}[H_2(SbW_9O_{33})(W_5O_{12})(Sb_2W_{29}O_{103})]$ • 115.5 H_2O): K, 3.18; Na, 2.72; Sb, 2.70; W, 58.51; Found: K, 3.64; Na, 2.79; Sb, 2.85; W, 59.00. EDX Anal. Element ratio found (calculated): K : Na : Sb : W = 11.1 (11.0) : 17.2 (16.0) : 2.5 (3.0) : 43 (43) **{Sb_3W_{43}}** (ATR-IR, cm^{-1}): 3380.9 (m), 1616.2 (s), 946.9 (s), 898.7 (s), 860.2 (s), 802.3 (s), 709.8 (s), 608.4)m), 552.8 (w), 469.3 (w), 425.2 (m), 304.7 (s).

2.2. Preparation of $K_8Na_{15}[H_{16}(Co(H_2O)_2)_{0.9}(Co(H_2O)_3)_2(W_{3.1}O_{14})(SbW_9O_{33})(Sb_2W_{30}O_{106})(H_2O)]$ • 53 H_2O {$Co_3Sb_3W_{42}$}

To a stirred solution of 360 µL $CoCl_2$ [1 M] in a mixture of 10 ml KOAc/NaOAc 5% (v/v) pH 5.5 acetate buffer and 5 ml H_2O **{Sb_3W_{43}}** (300 mg, 0.022 mmol) was added. The reaction mixture was stirred vigorously and heated to 40°C for 10 min. Filtration of the pink reaction mixture and evaporation at 20°C resulted in formation of pink plate shaped crystals of **{$Co_3Sb_3W_{42}$}** that started to form out of an initially appearing pink oil after four days. Yield: 180 mg, 65% based on W. Anal. Calcd. (%) for $K_8Na_{15}H_{140}Co_3Sb_3W_{42}O_{215}$($K_8Na_{15}[H_{16}(Co(H_2O)_2)_{0.9}(Co(H_2O)_3)_2(W_{3.1}O_{14})(SbW_9O_{33})(Sb_2W_{30}O_{106})(H_2O)]$ • 53 H_2O): K, 2.50; Na, 2.76; Co, 1.41; Sb, 2.92; W, 61.76; Found (XPS): K, 3.86; Na, 3.98; Co, 2.03; Sb, 2.01; W, 62.92. EDX Anal. Element ratio found (calculated): K : Na : Co : Sb : W = 8.1 (8.0) : 14.5 (15.0) : 3.2 (3.0) : 3.2 : (3.0) : 42 (42) **{$Co_3Sb_3W_{42}$}** (ATR-IR, cm^{-1}): 3363.5 (m), 1616.2 (w), 1556.4 (w), 1409.8 (w), 943.1 (s), 899.6 (s), 864.1 (s), 794.6 (s), 719.1 (s), 605.6 (m), 555.4 (m), 466.7 (m), 430.1 (s), 318.2 (s).

2.3. Preparation of $K_{20}Na_7[H_2(BiW_9O_{33})(W_5O_{12})(Bi_2W_{29}O_{103})]$ • 68 H_2O **{Bi_3W_{43}}**

To a hot (50 – 60°C) solution of Na_2WO_4 (1.32 g, 4 mmol) in 40 mL H_2O $Bi(OAc)_3$ (140 mg, 0.36 mmol), β-alanine (340 mg, 3.8 mmol) and KCl (300 mg, 4 mmol) were added. After adjustment of the hot reaction mixture to pH = 5.0 via dropwise addition of HCl [1 M], the solution was heated to 90°C for 60 min followed by filtration to yield a clear colorless solution. Slow evaporation of the reaction mixture at 25°C resulted in the formation of colorless needles of **{Bi_3W_{43}}**, which were removed from the mother liquor to avoid contamination by subsequently crystallizing block shaped crystals of **{Bi_2W_{22}}** as confirmed by SXRD measurements. Yield: 700 mg, 15% based on Bi. Anal. Calcd. (%) for $K_{20}Na_7H_{138}Bi_3W_{43}O_{216}$ ($K_{20}Na_7[H_2(BiW_9O_{33})(W_5O_{12})(Bi_2W_{29}O_{103})]$ • 68 H_2O): K, 5.98; Na, 1.23; Bi, 4.80; W, 60.48; Found: K, 4.31; Na, 1.34; Bi, 6.28; W, 57.80. EDX Anal. Element ratio found (calculated): K : Na : Bi : W = 20.4 (20.0) : 9.1 (7.0) : 2.3 (3.0) : 43 (43) **{Bi_3W_{43}}** (ATR-IR, cm^{-1}): 3404.1 (m), 1618.2 (w), 1558.3 (w), 1404.1 (w), 935.4 (s), 897.6 (s), 850.5 (s), 790.7 (s), 709.7 (s), 608.6 (m), 554.3 (w), 465.4 (m), 425.3 (s), 308.6 (s).

Table S1. Survey of polyoxotungstate crystal structures incorporating a full – or defect pentagonal unit according to *Scifinder* – and the ICSD database (January 2021).

Formula	Heteroatom	W nuclearity	Number of (defect) pentagonal units	Ref.
$[\{(W^{VI})W^{VI}_5O_{21}(H_2O)_5(CH_3COO)_{0.5}\}_{12}\{Mo^{V}_2O_4(CH_3COO)\}_{30}]^{48-}$	Mo^{V}	72	12	9
$[K_2@\{(B^{III}W^{VI}_{11}O_{39})_2(H_5Mo^{V}_{13}W^{VI}_2O_{48})\}]^{17-}$	B^{III}, Mo^{V}	24	2	10
$[Cs@\{(B^{III}W^{VI}_{11}O_{39})_4(H_{16}Mo^{V}_8W^{VI}_8O_{52})\}]^{27-}$	B^{III}, Mo^{V}	52	4	10
$[\{(W^{VI})W^{VI}_5O_{21}(SO_4)\}_{12}\{(Fe^{III}(H_2O))_{30}\}(SO_4)_{13}(H_2O)_{34}]^{32-}$	Fe^{III}	72	12	11
$\{H_{16}Co^{II}_8W^{VI}_{200}O_{660}(H_2O)_{40}\}^{88-}$	Co^{II}	200	16	12
$[H_{10}Ag^{I}_{18}Cl(Te^{IV}_3W^{VI}_{38}O_{134})_2]^{29-}$	Ag^{I}, Te^{IV}	76	2	13
$[H_2Te^{IV}_3W^{VI}_{43}O_{148}]^{24-}$	Te^{IV}	43	1	13
$[H_{12}W^{VI}_{48}O_{164}]^{28-}$	-	48	2	14
$[H_{20}W^{VI}_{56}O_{190}]^{24-}$	-	56	4	14
$[H_{12}W^{VI}_{92}O_{311}]^{58-}$	-	92	8	14
$[H_{34}W^{VI}_{119}Se^{IV}_8Fe^{III}_2O_{420}]^{54-}$	Fe^{III}, Se^{IV}	119	4	15
$[H_{10}Se^{IV}_2W^{VI}_{29}O_{103}]^{14-}$	Se^{IV}	29	1	16
$[H_{19}Co^{II}_{2.5}(W^{VI}_{3.5}O_{14})(Se^{IV}W^{VI}_9O_{33})(Se^{IV}_2W^{VI}_{30}O_{107})]^{17-}$	Co^{II}, Se^{IV}	42.5	1	16
$[H_4Co^{II}W^{VI}O(H_2O)_3(Se^{IV}_2W^{VI}_{26}O_{85})(Se^{IV}_3W^{VI}_{30}O_{107})_2]^{40-}$	Co^{II}, Se^{IV}	76	3	16
$[H_{14}Ni^{II}_2W^{VI}_2O_2Cl(H_2O)_3(Se^{IV}_2W^{VI}_{29}O_{103})(Se^{IV}_3W^{VI}_{30}O_{107})_2]^{43-}$	Ni^{II}, Se^{IV}	76	3	16
$\{M_2W^{VI}_nO_m(H_2O)_m(Se^{IV}_2W^{VI}_{29}O_{102})_4\}$	(M = Mn^{II}, Co^{II}, Ni^{II} or Zn^{II}, n = 2, m = 4; M = Cu^{II}, n = 3, m = 5)	116	4	16
$[H_{18}Cu^{II}_9Cl_3(H_2O)_{18}(Se^{IV}_2W^{VI}_{29}O_{102})_6]^{99-}$	Cu^{II}, Se^{IV}	174	6	16
**$[H_2(X^{III}W^{VI}_9O_{33})(W^{VI}_5O_{12})(X^{III}_2W^{VI}_{29}O_{103})]^{27-}$**	**X= Sb^{III}, Bi^{III}**	**43**	**1**	**this work**
**$[H_{16}(Co(H_2O)_2)_{0.9}(Co(H_2O)_3)_2(W_{3.1}O_{14})(SbW_9O_{33})(Sb_2W_{30}O_{106})(H_2O)]^{23-}$**	**Co^{II}, Sb^{III}**	**42**	**1**	**this work**

Table S2. Survey of existing unsubstituted tungstoantimonate-and bismuthate crystal structures according to the ICSD database (January 2021).

Formula	Sb nuclearity	W nuclearity	Ref.
$[Sb^{V}W_{18}O_{60}(OH)_2]^{9-}$	1	18	17
$[Sb^{III}W_9O_{33}]^{9-}$	1	9	1
$[Sb^{III}{}_2W_{22}O_{74}(OH)_2]^{12-}$	2	22	1
$[Na_2Sb^{III}{}_8W_{36}O_{132}(H_2O)_4]^{22-}$	8	36	1
$[H_{12}Sb^{V}{}_6W_4O_{36}]^{6-}$	6	4	18
$[Sb^{V}W_6O_{24}]^{7-}$	1	6	19
$[H_6Sb^{III}{}_2W_{21}O_{73}]^{8-}$	2	21	20
$\{[Sb^{III}{}_2(WO_2)_2(B\text{-}\beta\text{-}Sb^{III}W_9O_{33})_2][(WO_2)_2(WO_3)_2(B\text{-}\beta\text{-}Sb^{III}W_9O_{33})_2]\}^{14-}$	6	42	21
$[Bi^{III}{}_2W_{22}O_{76}]^{\mathbf{14-}}$	2	22	22
$[H_{(2-x)}Bi^{III}{}_2W_{20}O_{70}(HWO_3)_x]^{12-}$	2	20	23
$[(Na(H_2O)_2)_6(\alpha\text{-}Bi^{III}W_9O_{33})_2]^{12-}$	2	18	24
$[Bi^{III}(H_2W_{12}O_{42})]^{7-}$	1	12	25
$[H_2Bi^{III}W_{18}O_{60}]^{7-}$	1	18	26
$[H_3Bi^{III}W_{18}O_{60}]^{6-}$	1	18	27
$\mathbf{[H_2(Sb^{III}W_9O_{33})(W_5O_{12})(Sb^{III}{}_2W_{29}O_{103})]^{27-}}$	**3**	**43**	**this work**

3. IR-spectra

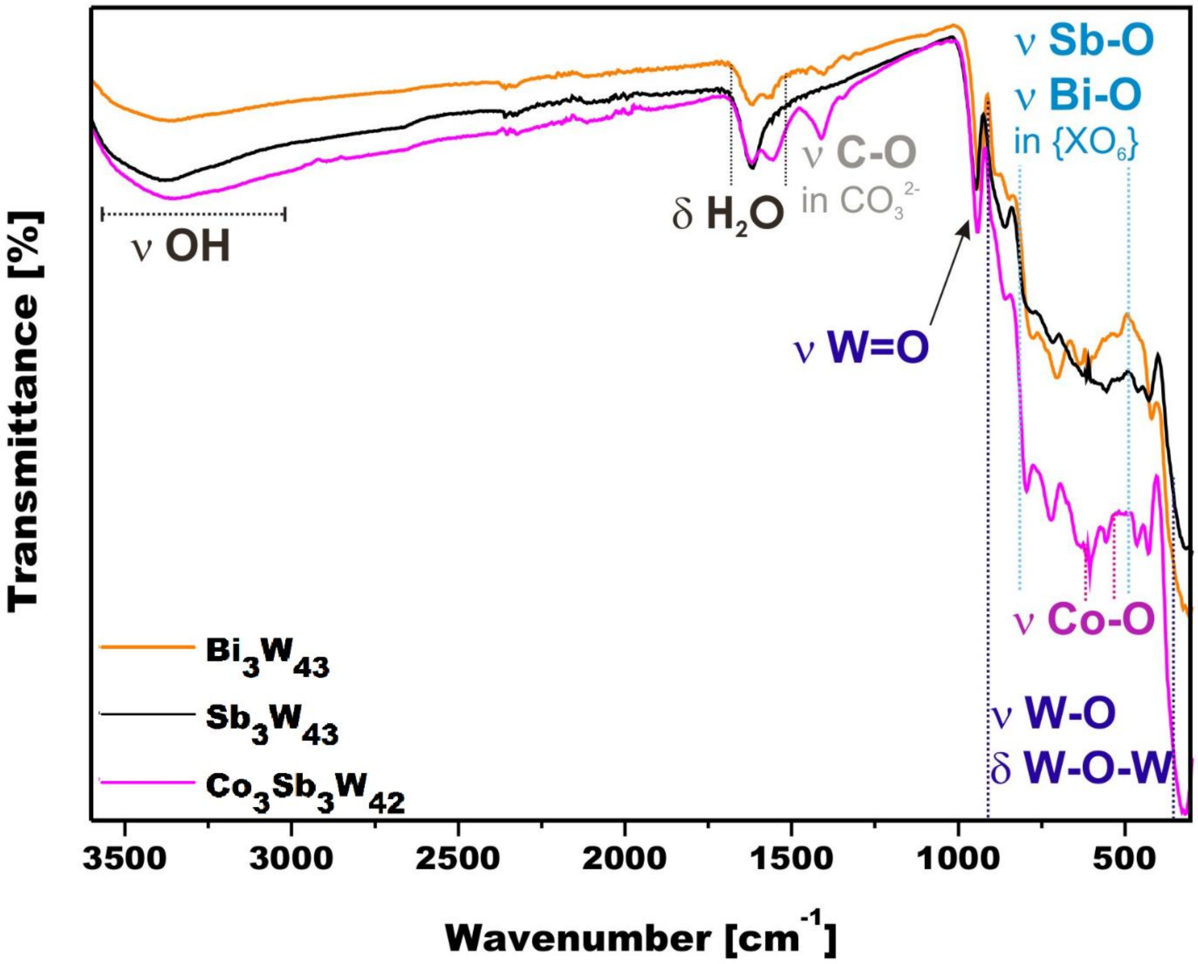

Figure S1. IR-spectra of **{X_3W_{43}}** (X = Bi^{III}, Sb^{III}) and **{$Co_3Sb_3W_{42}$}** from 4000 – 300 cm^{-1}.

Table S3. Attribution and positions of the bands observed in the IR-spectra of **{X_3W_{43}}** (X = Bi^{III}, Sb^{III}) and **{$Co_3Sb_3W_{42}$}.**

POT	position/range [cm^{-1}]	attribution	intensity
**Bi_3W_{43}**	3404.1	$^{\nu}$OH	m
	1618.2, 1558.3	$^{\delta}H_2O$	w
	935.4	$^{\nu}$W=O	s
	912.2 - 365	$^{\nu}$W=O, $^{\delta}$W-O-W	s
	835 - 495	$^{\nu}$Bi-O	s
**Sb_3W_{43}**	3380.9	$^{\nu}$OH	m
	1616.2	$^{\delta}H_2O$	s
	946.9	$^{\nu}$W=O	s
	912.2 - 365	$^{\nu}$W=O, $^{\delta}$W-O-W	s
	835 - 495	$^{\nu}$Sb-O	s
**$Co_3Sb_3W_{42}$**	3363.5	$^{\nu}$OH	m

	1616.2, 1556.4	$^{\delta}H_2O$	w
	1409.8	$^{\nu}$C-O in CO_3^{2-}	w
	943.1	$^{\nu}$W=O	s
	912.2 - 365	$^{\nu}$W=O, $^{\delta}$W-O-W	s
	835 - 495	$^{\nu}$Sb-O	s
	605.6, 555.4	$^{\nu}$Co-O	m

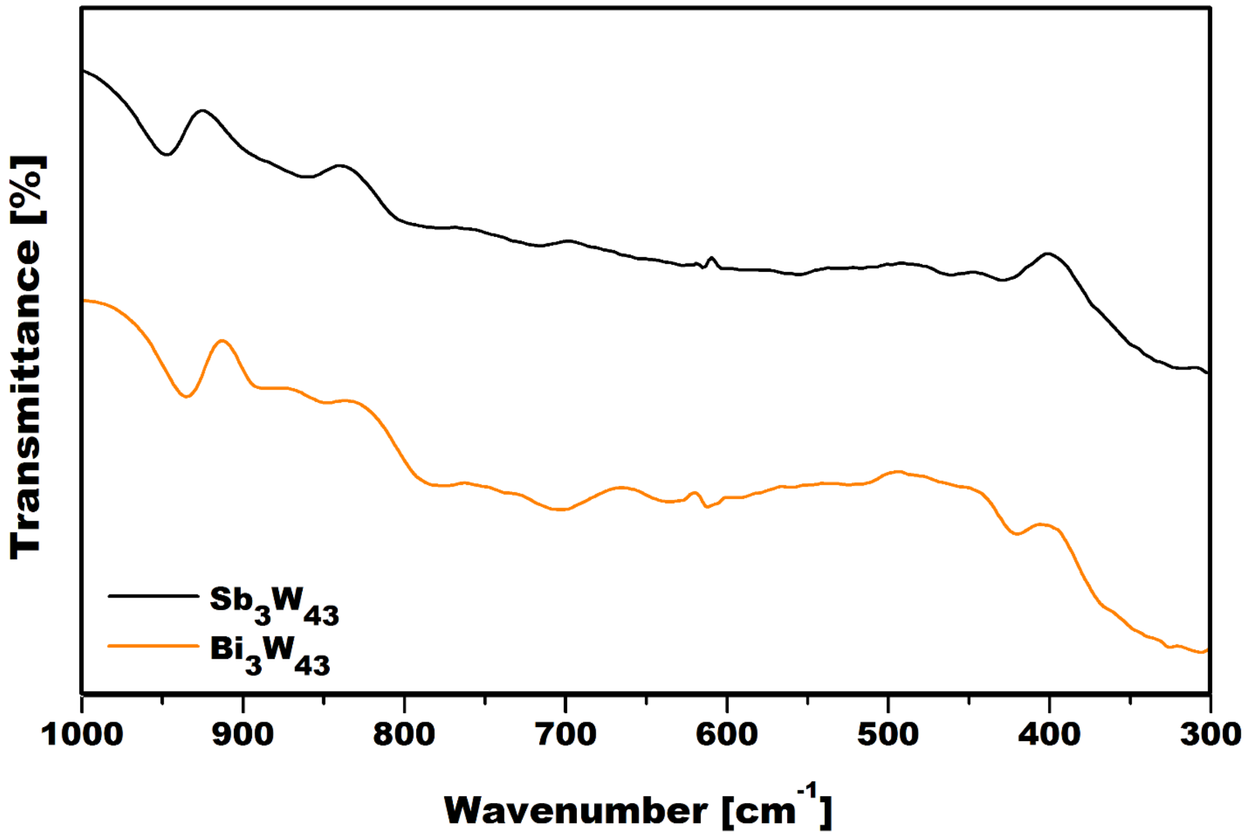

Figure S2. Superimposed IR-spectra of **{Sb_3W_{43}}** and **{Bi_3W_{43}}** in the tungsten fingerprint region from 1000 – 300 cm^{-1} highlighting the isostructural composition of the POT frameworks.

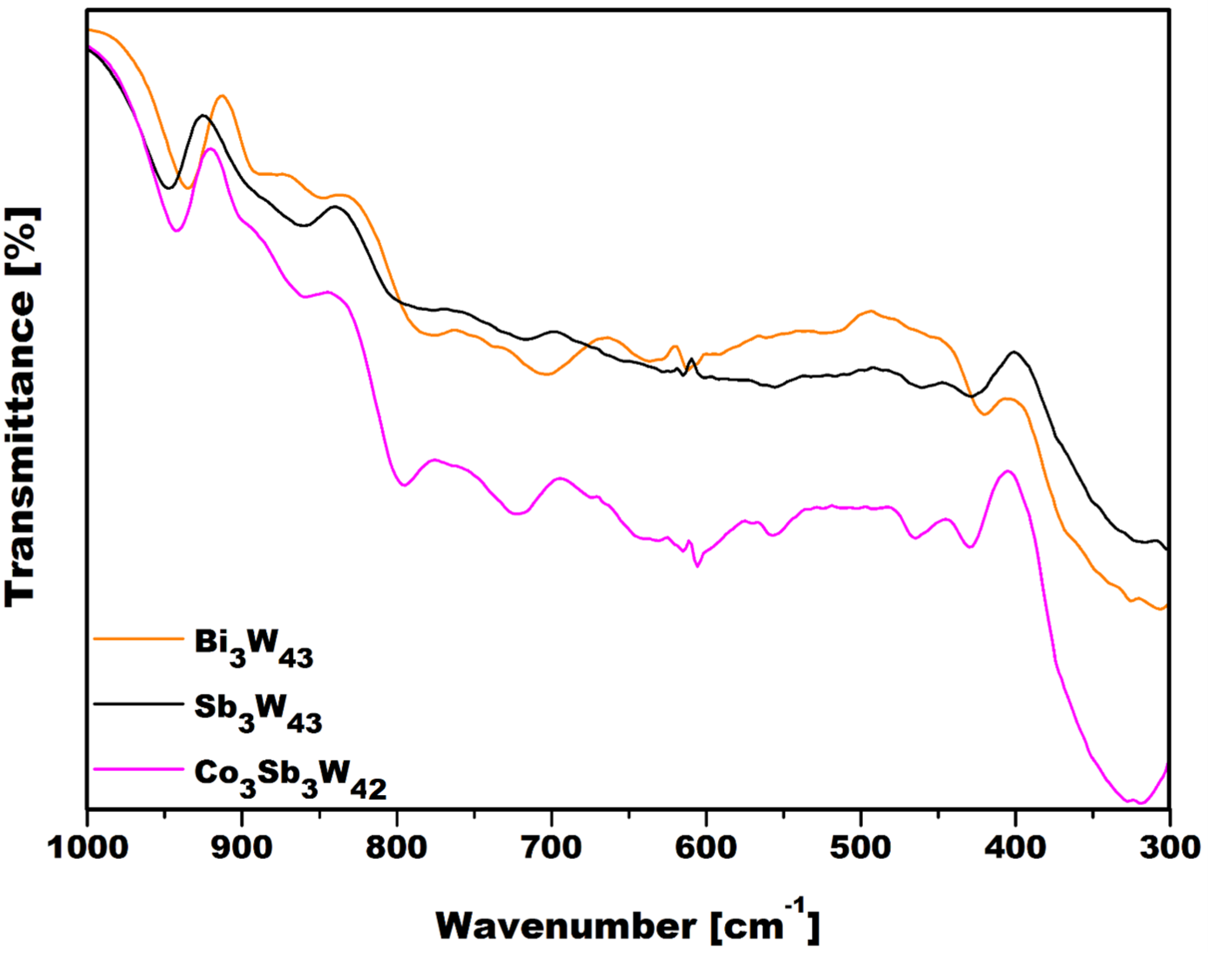

Figure S3. Superimposed IR-spectra of **{Sb_3W_{43}}**, **{Bi_3W_{43}}** and **{$Co_3Sb_3W_{42}$}** in the tungsten fingerprint region from 1000-300 cm^{-1}.

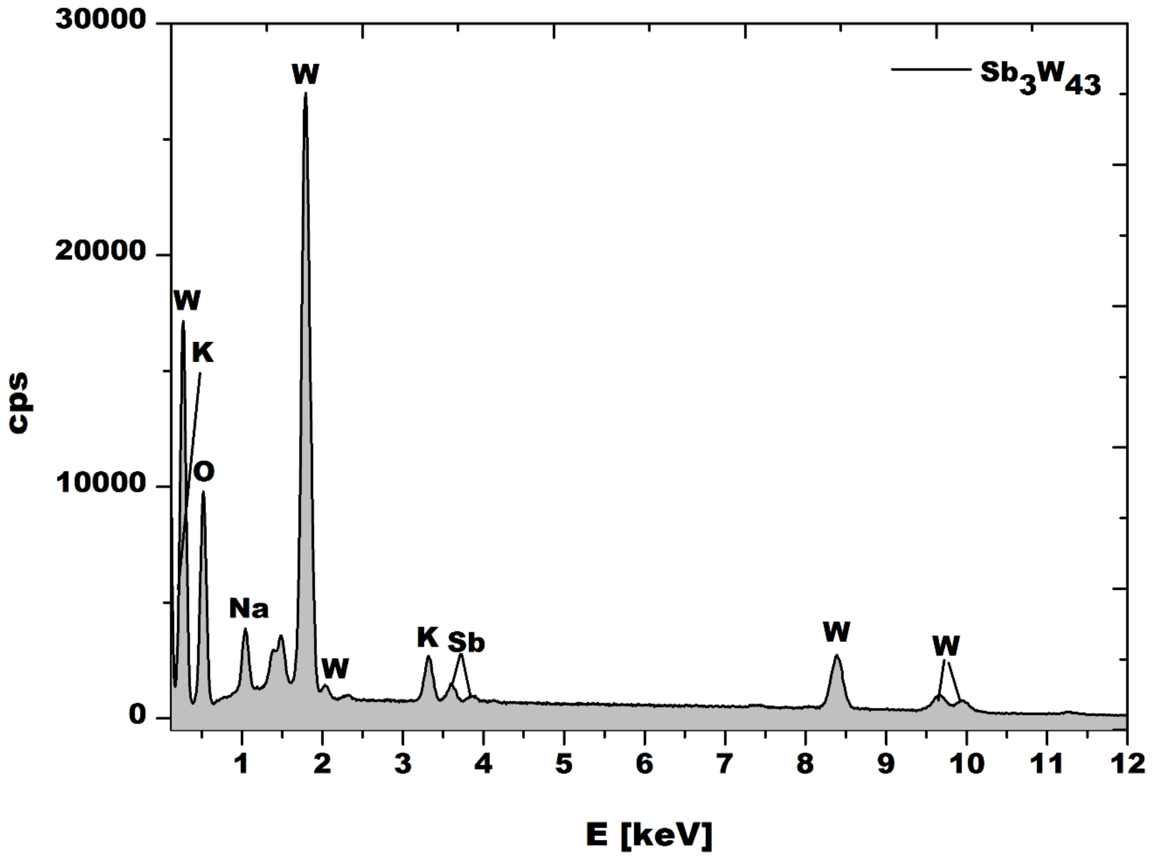

Figure S4. Energy dispersive X-ray analysis (EDX) spectrum of **{Sb_3W_{43}}** (cps = counts per second).

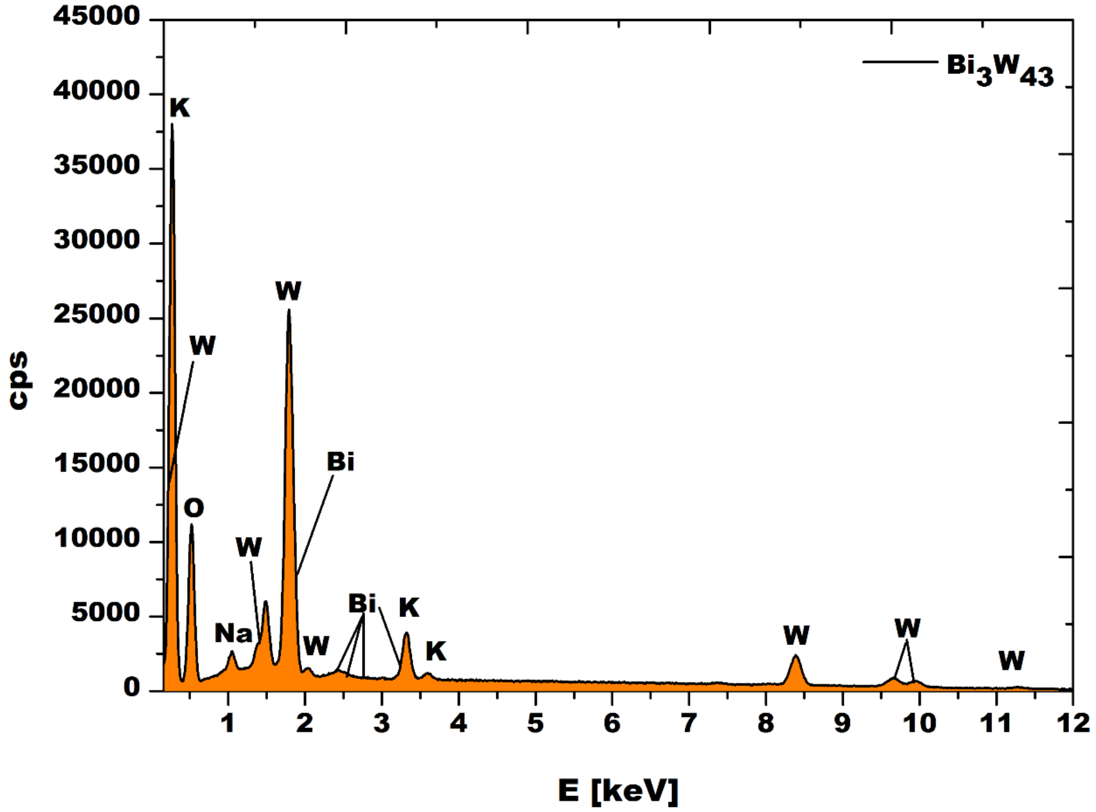

Figure S5. Energy dispersive X-ray analysis (EDX) spectrum of **{Bi_3W_{43}}** (cps = counts per second).

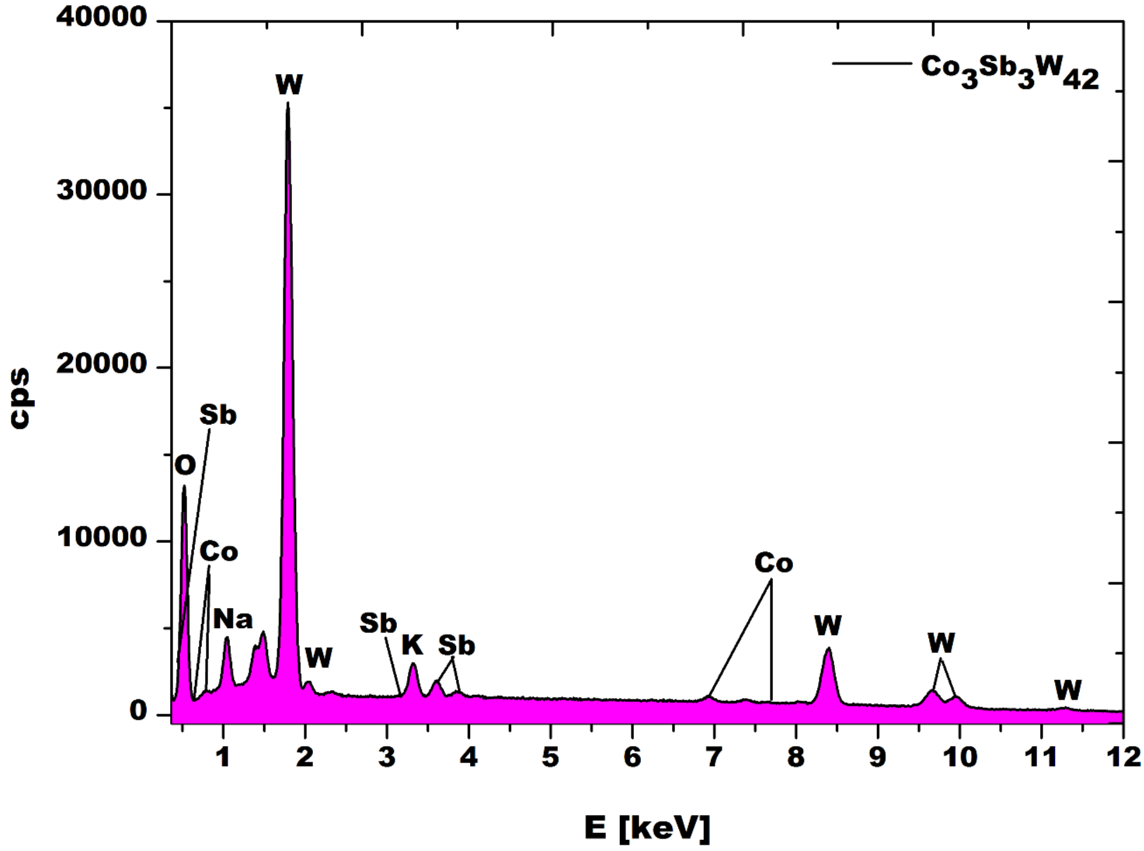

Figure S6. Energy dispersive X-ray analysis (EDX) spectrum of **{$Co_3Sb_3W_{42}$}** (cps = counts per second).

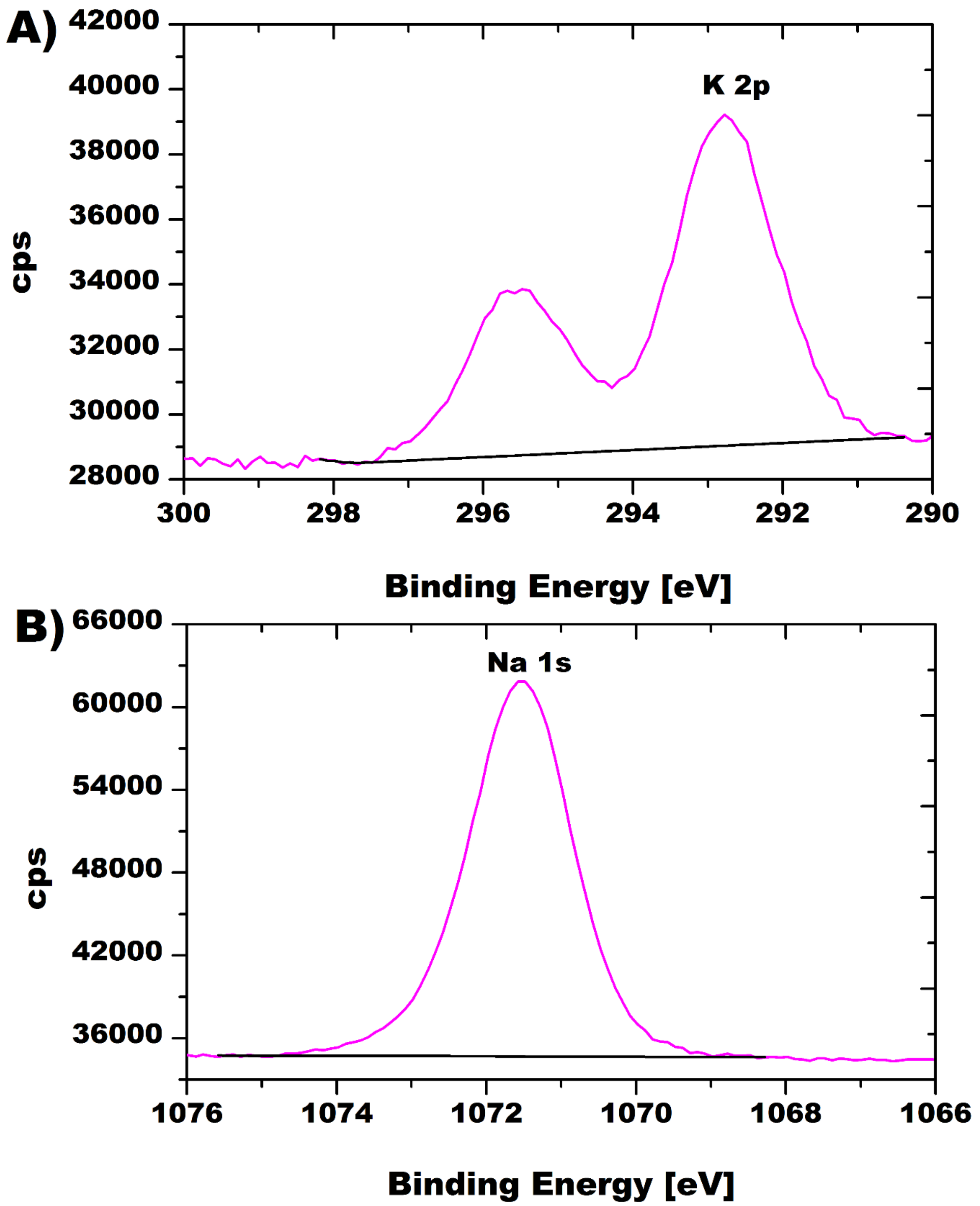

Figure S7. High resolution X-ray photoelectron spectra (XPS) of **{$Co_3Sb_3W_{42}$}** in the **A)** K 2p - and **B)** Na 1s range (cps = counts per second).

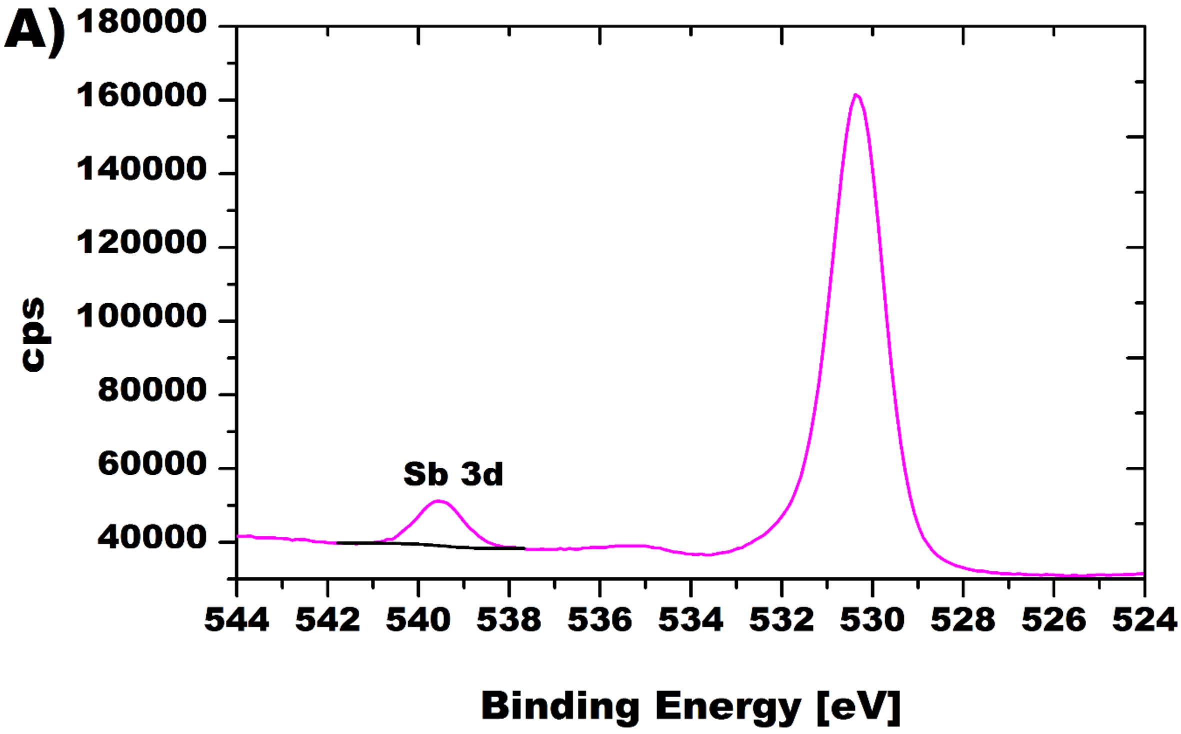

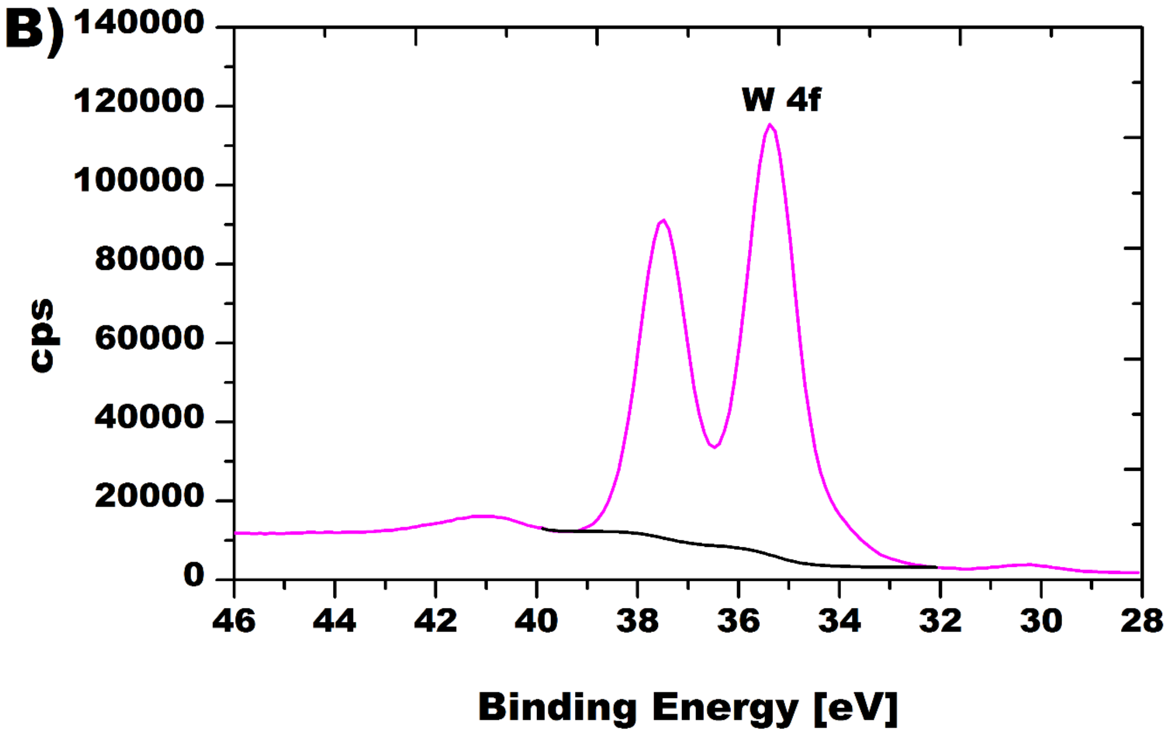

Figure S8. High resolution X-ray photoelectron spectra (XPS) of **{$Co_3Sb_3W_{42}$}** in the **A)** Sb 3d - and **B)** W 4f range (cps = counts per second).

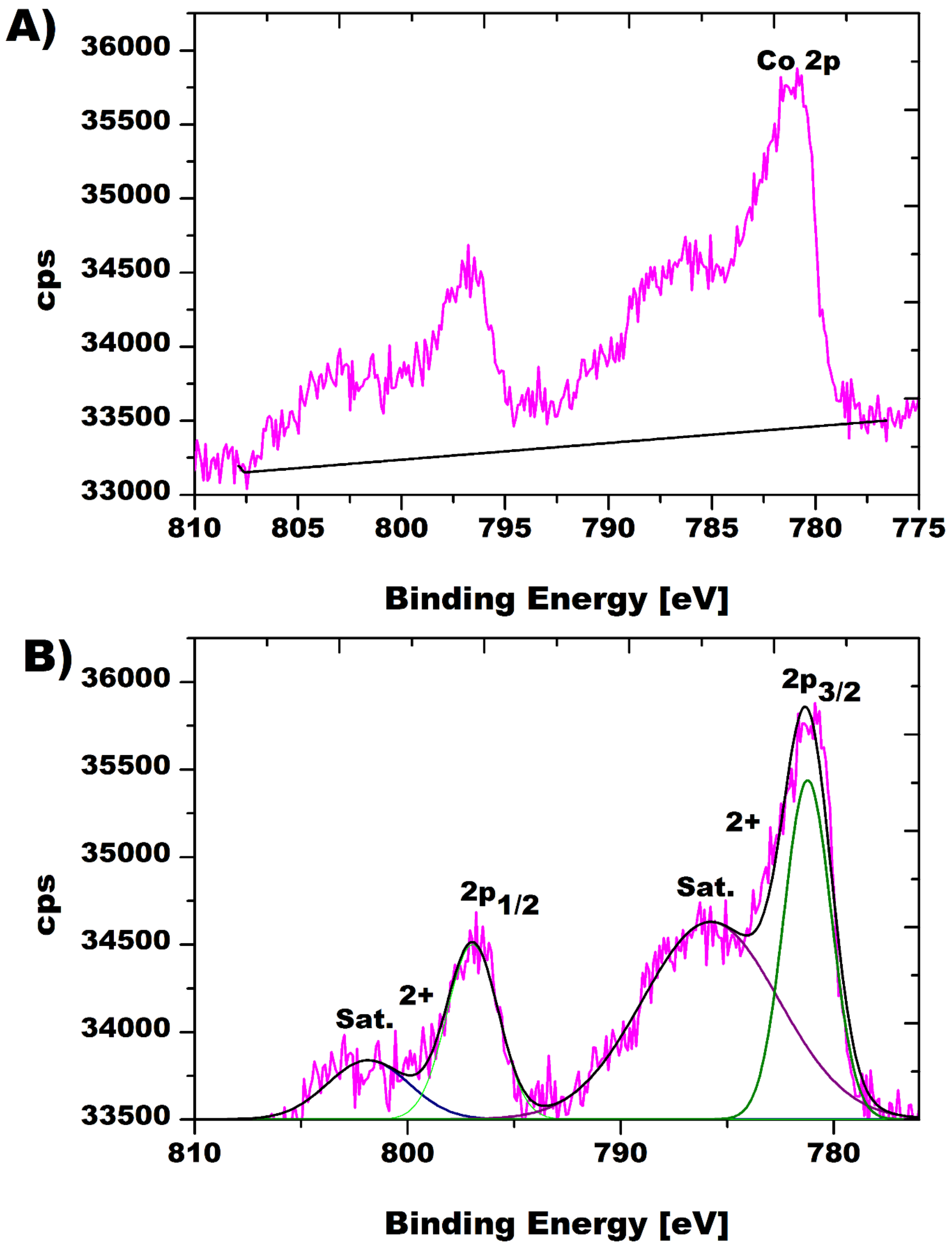

Figure S9. High resolution X-ray photoelectron spectra (XPS) of **{$Co_3Sb_3W_{42}$}** in the **A)** Co 2p range and **B)** Gaussian fit revealing the Co 2p core levels (cps = counts per second).

4. Thermogravimetric Analysis

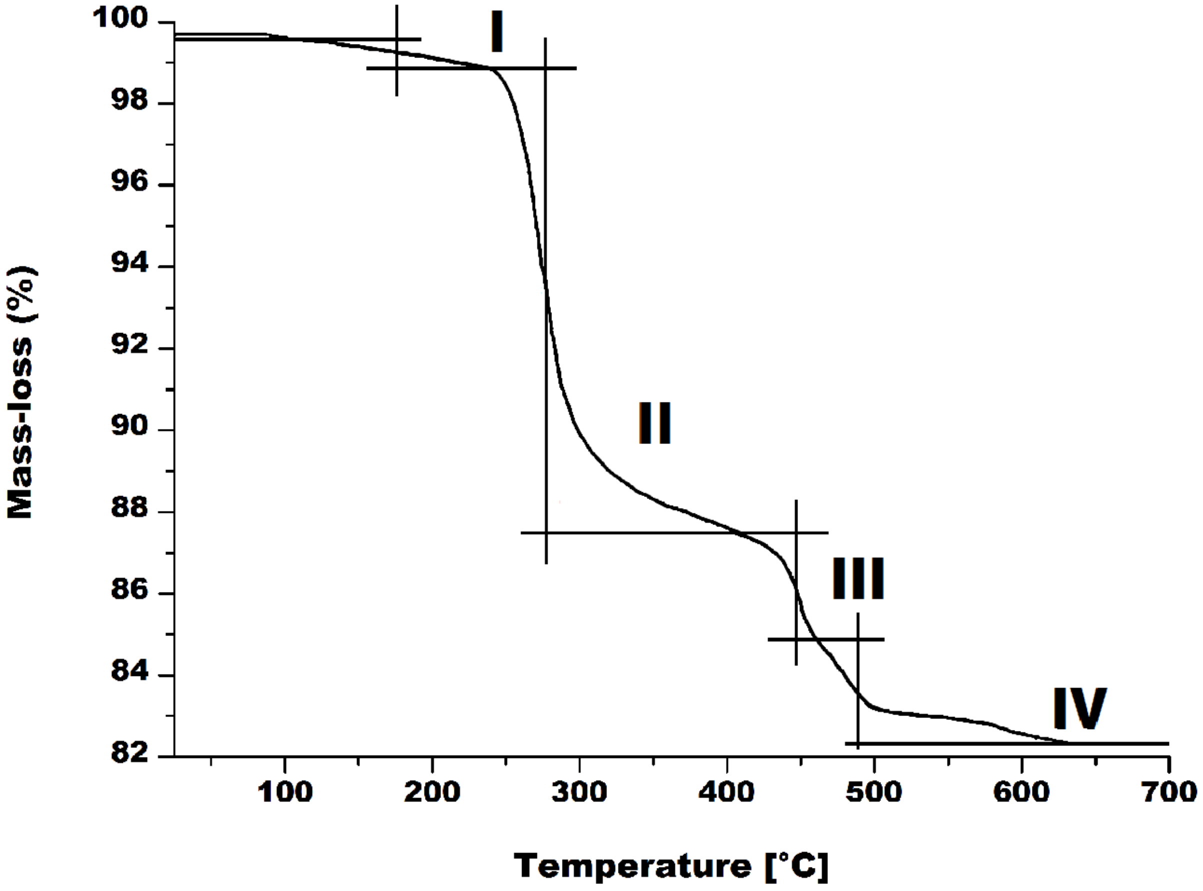

Figure S10. Thermogravimetric curve of $K_{11}Na_{16}[H_2(SbW_9O_{33})(W_5O_{12})(Sb_2W_{29}O_{103})]$ • 115.5 H_2O **{Sb_3W_{43}}**. A discrepancy of < 0.5% in the mass loss at 25°C can be attributed to the isothermal equilibration performed at 25°C leading to possible initial loss of moisture.

Table S4. TGA results for $K_{11}Na_{16}[H_2(SbW_9O_{33})(W_5O_{12})(Sb_2W_{29}O_{103})]$ • 115.5 H_2O **{Sb_3W_{43}}**.

Step	T, °C	mass-loss, %	number of H_2O molecules corresponding to mass-loss
I	25-150	0.73	4.7
II	150-250	10.96	78
III	250-400	2.57	16.7
IV	400-700	2.47	16.1

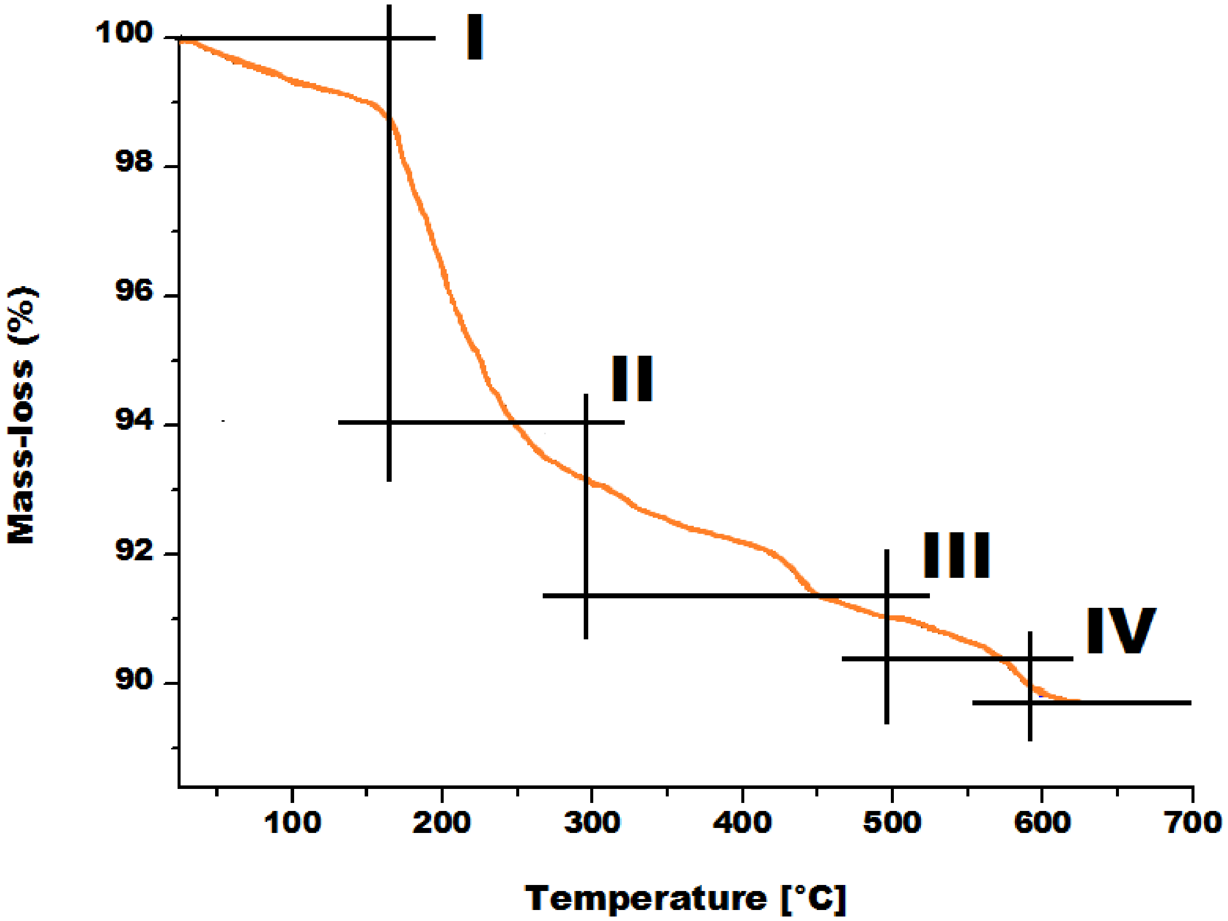

Figure S11. Thermogravimetric curve of $K_{20}Na_7[H_2(BiW_9O_{33})(W_5O_{12})(Bi_2W_{29}O_{103})]$ • 68 H_2O **{Bi_3W_{43}}**.

Table S5. TGA results for $K_{20}Na_7[H_2(BiW_9O_{33})(W_5O_{12})(Bi_2W_{29}O_{103})]$ • 68 H_2O **{Bi_3W_{43}}**.

Step	T, °C	mass-loss, %	number of H_2O molecules corresponding to mass-loss
I	25-250	0.93	6
II	250-450	5.72	40
III	450-550	2.03	14
IV	550-700	1.23	8

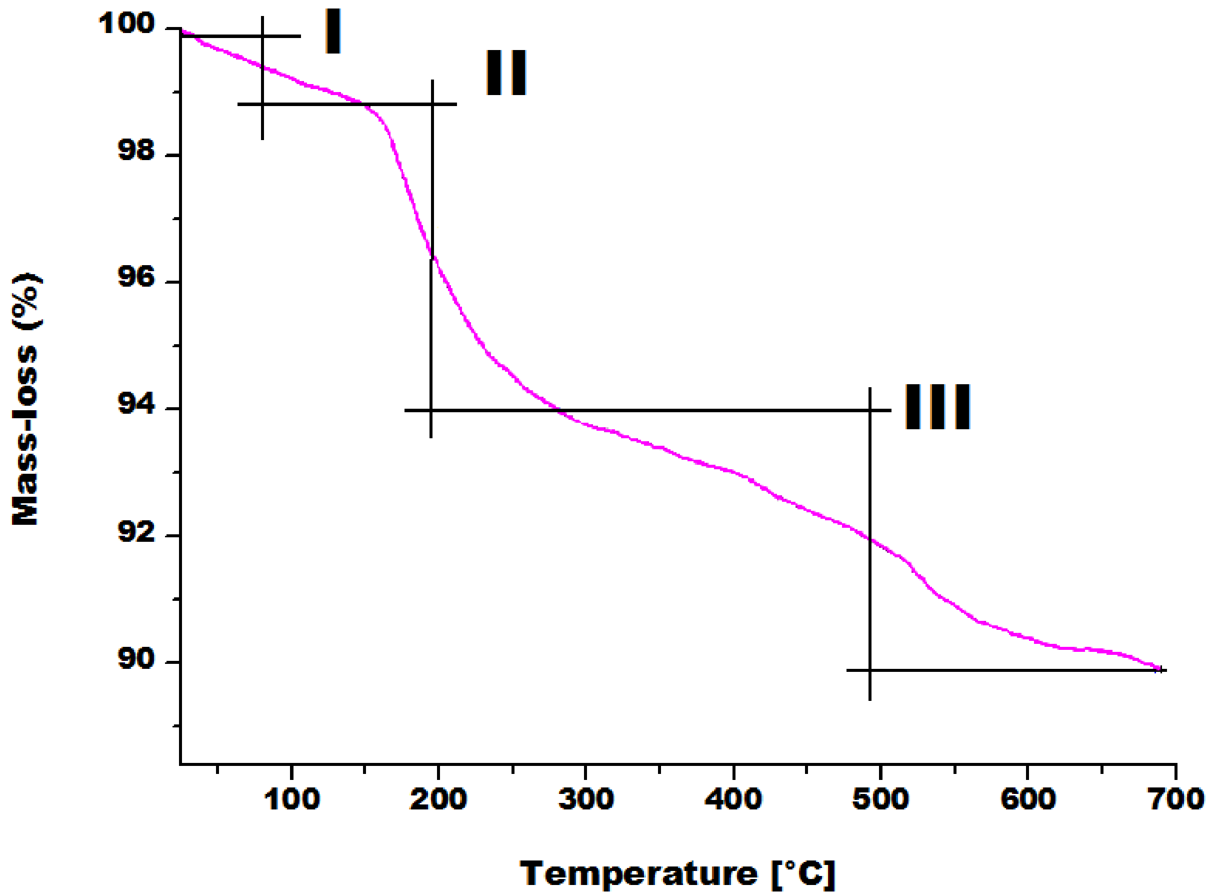

Figure S12. Thermogravimetric curve of $K_8Na_{15}[H_{16}(Co(H_2O)_2)_{0.9}(Co(H_2O)_3)_2(W_{3.1}O_{14})(SbW_9O_{33})(Sb_2W_{30}O_{106})(H_2O)] \cdot 53\ H_2O$ **{Co$_3$Sb$_3$W$_{42}$}**.

Table S6. TGA results for $K_8Na_{15}[H_{16}(Co(H_2O)_2)_{0.9}(Co(H_2O)_3)_2(W_{3.1}O_{14})(SbW_9O_{33})(Sb_2W_{30}O_{106})(H_2O)] \cdot 53\ H_2O$ **{Co$_3$Sb$_3$W$_{42}$}**.

Step	T, °C	mass-loss, %	number of H_2O molecules corresponding to mass-loss
I	25-150	1.03	7
II	150-250	4.57	30
III	250-700	3.86	25

5. Single-Crystal X-ray Diffraction

Table S7. Experimental parameter and CCDC-Code.

Sample	Machine	Source	Temp.	Detector Distance	Time/ Frame	#Frames	Frame width	CCDC
			[K]	[mm]	[s]		[°]	
**{Sb_3W_{43}}**	Bruker D8	Mo	100	35	4	958	0.5	2070328
**{$Co_3Sb_3W_{42}$}**	Bruker D8	Cu	110	30	5	971	1	2070860

Table S8. Sample and crystal data of **{Sb_3W_{43}}.**

**Chemical formula**	$K_{11}Na_{16}H_{233}Sb_3W_{43}O_{263}$	**Crystal system**	triclinic	
**Formula weight [g/mol]**	13511.00	**Space group**	***P1̅***	
**Temperature [K]**	100.0	**Z**	2	
**Measurement method**	φ and ω scans	**Volume [Å^3]**	10781.7(6)	
**Radiation (Wavelength [Å])**	MoK*α* (λ = 0.71073)	**Unit cell dimensions [Å] and [°]**	17.8217(6)	76.3923(12)
**Crystal size / [mm^3]**	0.394 × 0.35 × 0.132		20.7584(7)	89.7834(14)
**Crystal habit**	clear colorless block		30.0523(10)	86.2356(13)
**Density (calculated) / [g/cm^3]**	3.424	**Absorption coefficient / [mm^{-1}]**	23.515	
**Abs. correction Tmin**	0.1258	**Abs. correction Tmax**	0.9602	
**Abs. correction type**	multi-scan	**F(000) [e$^-$]**	9498.0	

Table S9. Data collection and structure refinement of **{Sb_3W_{43}}.**

**Index ranges**	-20 ≤ h ≤ 20, -23 ≤ k ≤ 24, -34 ≤ l ≤ 34	**Theta range for data collection [°]**	4.36 to 48.812	
**Reflections number**	140871	**Data / restraints / parameters**	35469/13/1157	
**Refinement method**	Least squares	**Final R indices**	all data	R_1 = 0.1060, wR_2 = 0.2771
**Function minimized**	Σ w(F_o^2 - F_c^2)^2		I>2σ(I)	R_1 = 0.0961, wR_2 = 0.2642
**Goodness-of-fit on F^2**	1.101	**Weighting scheme**	w=1/[σ^2(F_o^2)+(0.1891P)^2+21.3435P]	
**Largest diff. peak and hole [e Å^{-3}]**	6.70/-4.89		where P=(F_o^2+2F_c^2)/3	

Table S10. Sample and crystal data of **{$Co_3Sb_3W_{42}$}.**

**Chemical formula**	$K_8Na_{15}H_{140}Co_3Sb_3W_{42}O_{215}$	**Crystal system**	triclinic	
**Formula weight [g/mol]**	12501.97	**Space group**	$P\bar{1}$	
**Temperature [K]**	112.0	**Z**	2	
**Measurement method**	φ and ω scans	**Volume [$Å^3$]**	9769(3)	
**Radiation (Wavelength [Å])**	CuK*α* (λ = 1.54178)	**Unit cell dimensions [Å] and [°]**	19.222(3)	65.141(8)
**Crystal size / [mm^3]**	0.07 × 0.04 × 0.03		24.006(4)	86.151(12)
**Crystal habit**	clear pink plate		24.913(6)	70.094(9)
**Density (calculated) / [g/cm^3]**	3.979	**Absorption coefficient / [mm^{-1}]**	51.827	
**Abs. correction Tmin**	0.039	**Abs. correction Tmax**	0.1256	
**Abs. correction type**	multi-scan	**F(000) [e^-]**	10106.0	

Table S11. Data collection and structure refinement of **{$Co_3Sb_3W_{42}$}.**

**Index ranges**	-12 ≤ h ≤ 12, -15 ≤ k ≤ 15, -16 ≤ l ≤ 16	**Theta range for data collection [°]**	4.322 to 61.848	
**Reflections number**	30819	**Data / restraints / parameters**	6056/233/1249	
**Refinement method**	Least squares	**Final R indices**	all data	R_1 = 0.1039, wR_2 = 0.2543
**Function minimized**	$\Sigma\ w(F_o^2 - F_c^2)^2$		I>2σ(I)	R_1 = 0.0910, wR_2 = 0.2397
**Goodness-of-fit on F^2**	1.050	**Weighting scheme**	$w=1/[\sigma^2(F_o^2)+(0.1581P)^2+1711.4846P]$	
**Largest diff. peak and hole [e $Å^{-3}$]**	5.09/-1.80		where $P=(F_o^2+2F_c^2)/3$	

6. Powder X-ray Diffraction

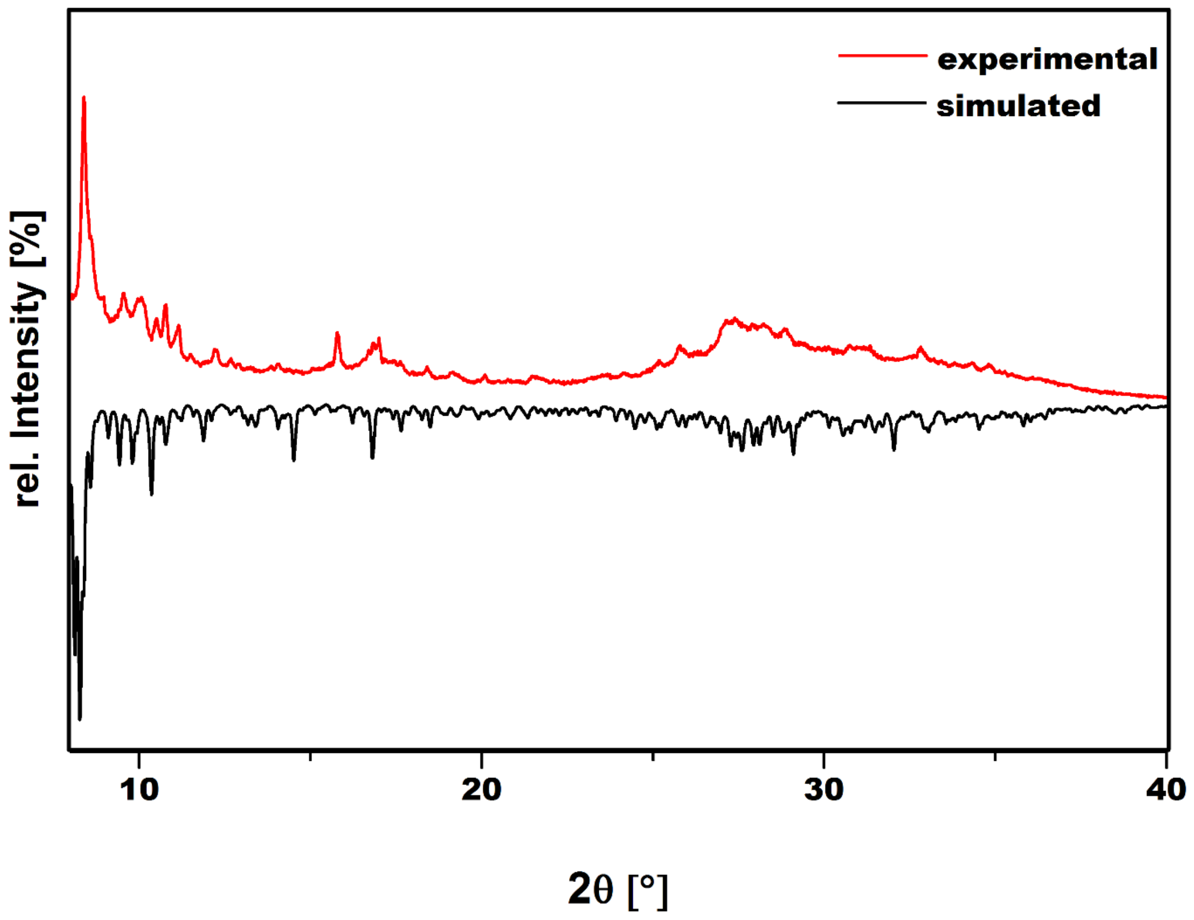

Figure S13. Comparison of the experimental and simulated PXRD patterns of $K_{11}Na_{16}[H_2(SbW_9O_{33})(W_5O_{12})(Sb_2W_{29}O_{103})]$ • 115.5 H_2O **{Sb_3W_{43}}**. Note that differences between the simulated and the experimental PXRD patterns may be due to factors such as scanning speed, preferred orientation, and efflorescence of the crystals, which lose solvent molecules further leading to the collapse of the lattice.

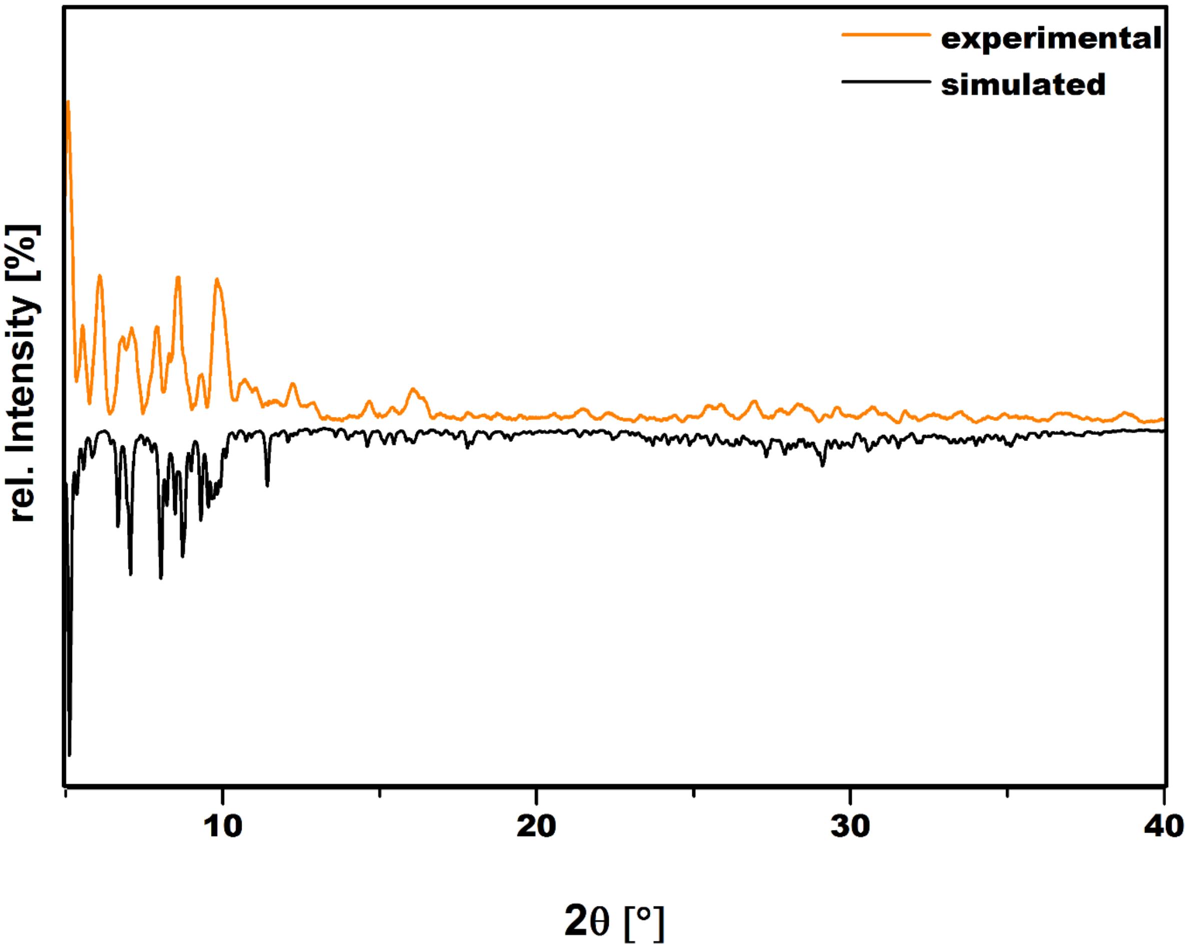

Figure S14. Comparison of the experimental and simulated PXRD patterns of $K_{20}Na_7[H_2(BiW_9O_{33})(W_5O_{12})(Bi_2W_{29}O_{103})]$ • 68 H_2O **{Bi_3W_{43}}**. The simulated pattern was obtained from the SXRD data collected on a needle shaped single-crystal of **{Bi_3W_{43}}**. Despite the weakly diffracting nature of the crystals, their quality was sufficient to obtain a simulated PXRD pattern. Note that differences between the simulated and the experimental PXRD patterns may be due to factors such as scanning speed, preferred orientation, and efflorescence of the crystals, which lose solvent molecules further leading to the collapse of the lattice.

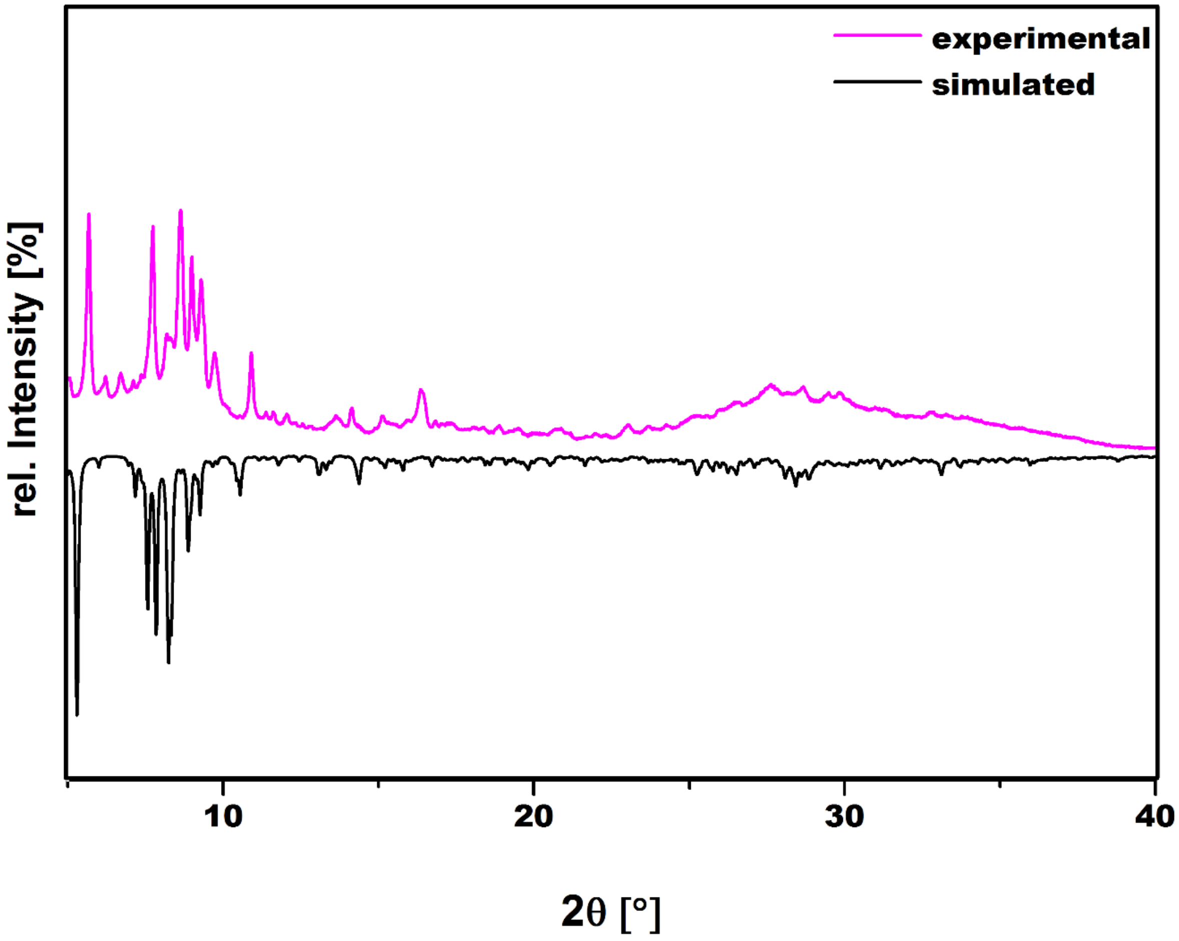

Figure S15. Comparison of the experimental and simulated PXRD patterns of $K_8Na_{15}[H_{16}(Co(H_2O)_2)_{0.9}(Co(H_2O)_3)_2(W_{3.1}O_{14})(SbW_9O_{33})(Sb_2W_{30}O_{106})(H_2O)]$ • 53 H_2O **{$Co_3Sb_3W_{42}$}**. Note that differences between the simulated and the experimental PXRD patterns may arise from various factors such as scanning speed, preferred orientation, and efflorescence of the crystals, which lose solvent molecules further leading to the collapse of the lattice.

7. UV/Vis Spectroscopy

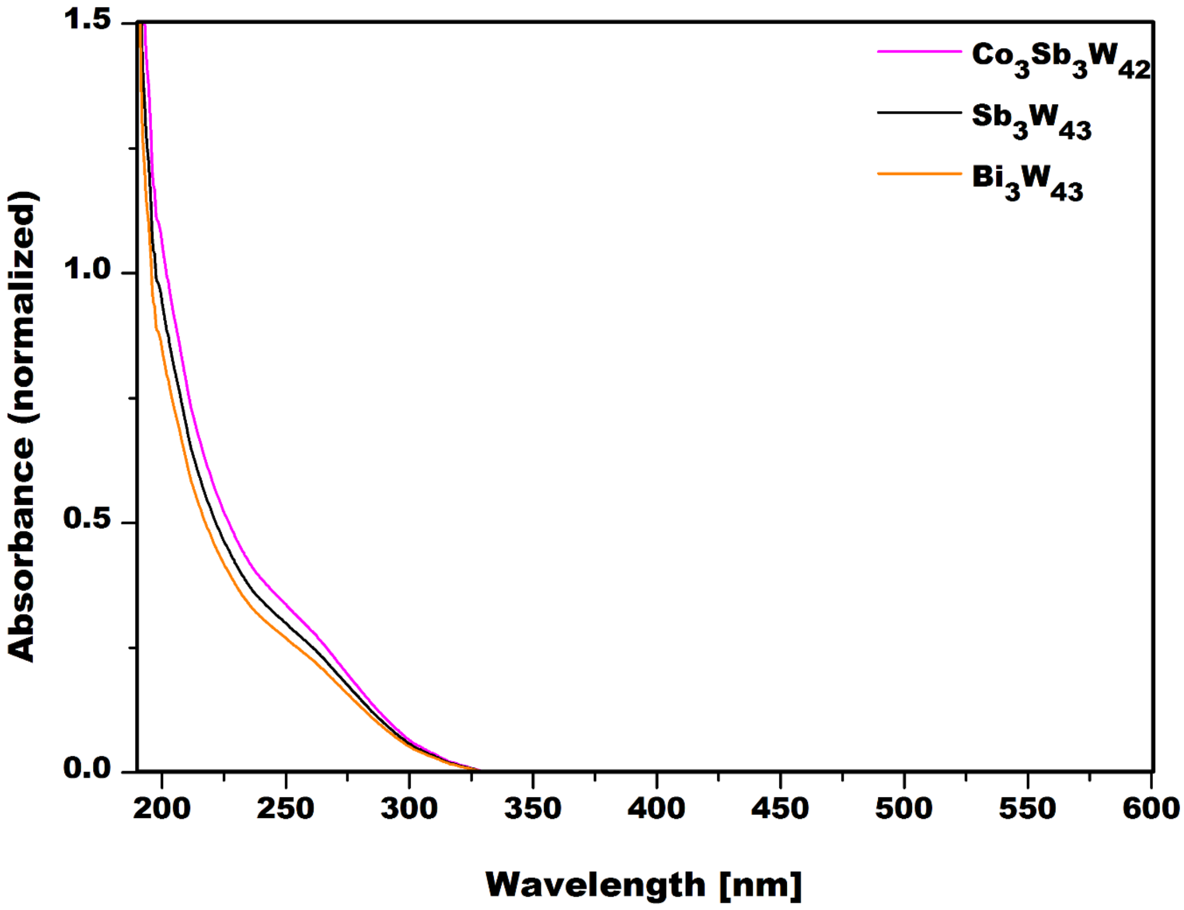

Figure S16. UV/Vis-spectra of [10 μM] **{X_3W_{43}}** and **{$Co_3Sb_3W_{42}$}** in H_2O (pH = 6.8 via HCl [1 M]) showing the typical O→W ligand−to−metal charge−transfer with absorption maximum at ~205 nm and a shoulder at ~250 nm.

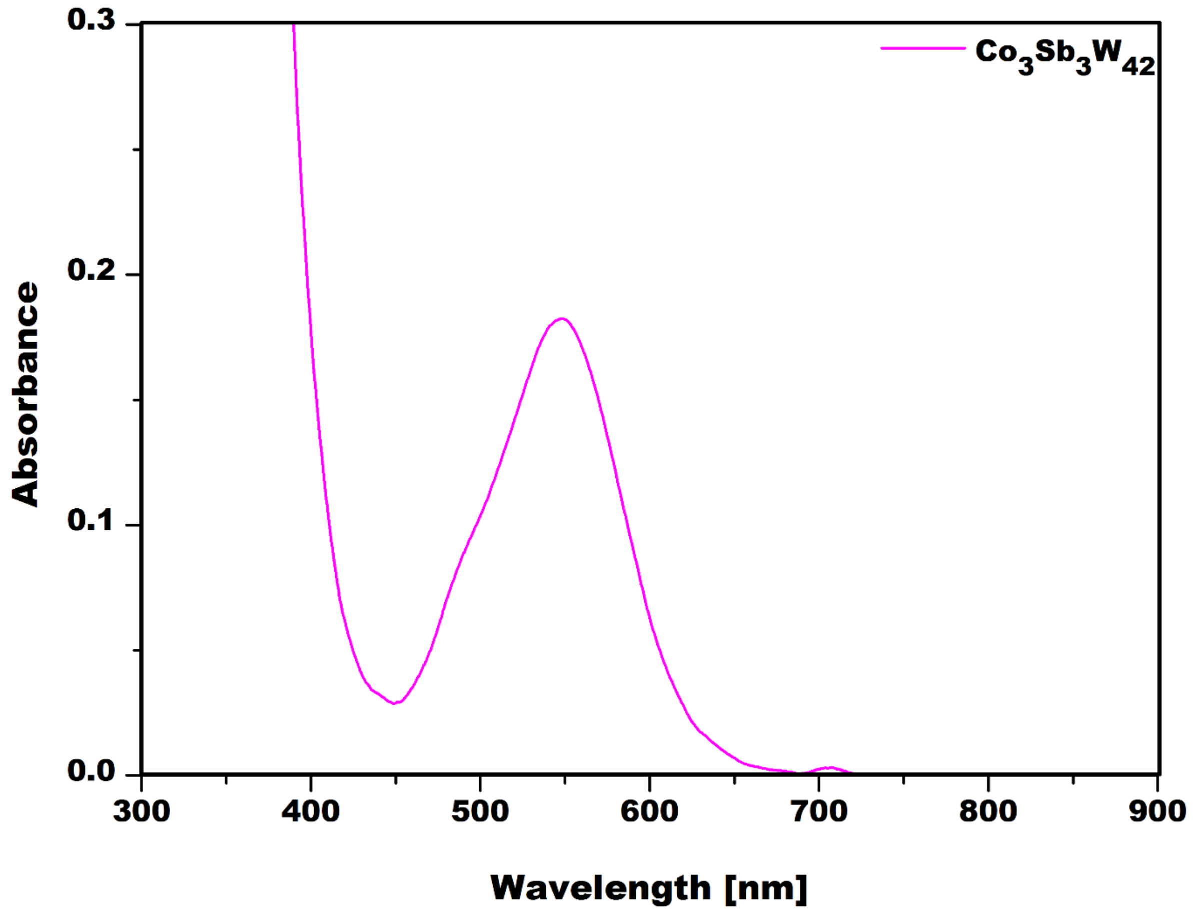

Figure S17. UV/Vis-spectrum of [3 mM] **{$Co_3Sb_3W_{42}$}** in H_2O (pH = 6.8 via HCl [1 M]) showing the absorption in the visible region at ~548 nm typically attributed to the d – d transitions in octahedrally coordinated Co(II) species.

8. Catalysis

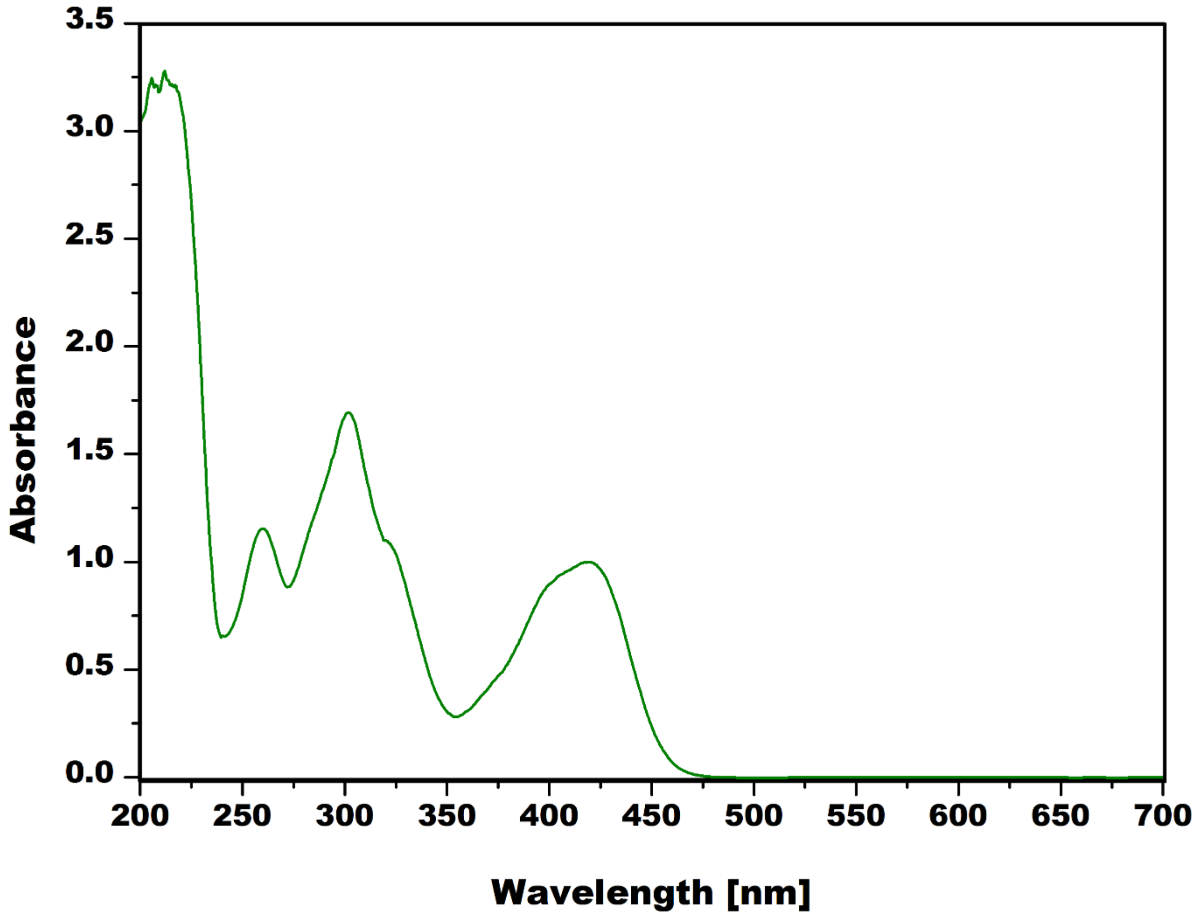

Figure S18. UV/Vis-spectrum of an aqueous [1 mM] stock solution containing $[Fe^{III}(CN)_6]^{3-}$ displaying a series of absorption bands at 272, 309, 325, and 420 nm, corresponding to a charge transition band and the $^2T_{1g} \rightarrow {}^2E_{1g}$, $^2T_{2u} \rightarrow {}^2T_{2g}$, $^2T_{2g} \rightarrow {}^2A_{1g}$, and $^2T_{1g} \rightarrow {}^2T_{2g}$ transitions, respectively. [28]

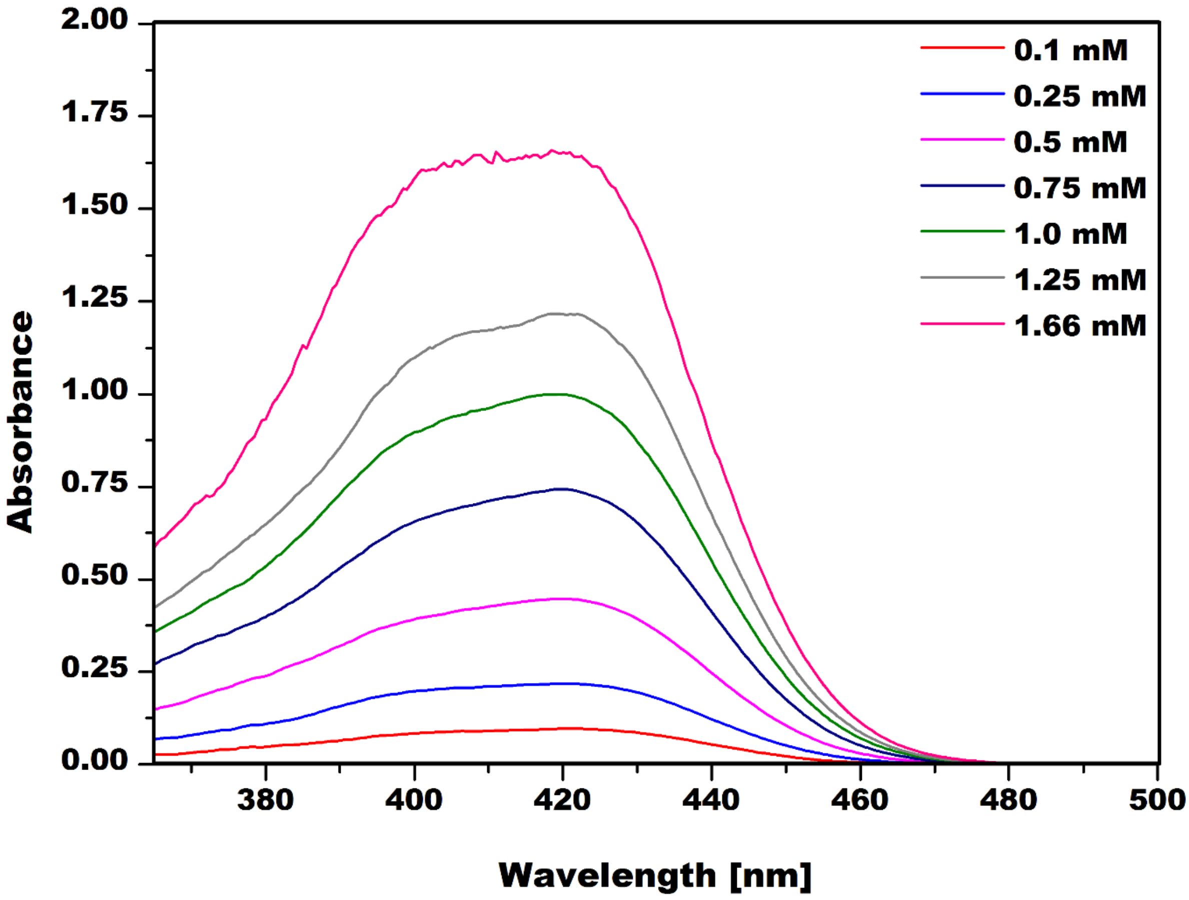

Figure S19. UV/Vis-spectra of $[Fe^{III}(CN)_6]^{3-}$ stock solutions with different concentrations displaying the characteristic absorption at 420 nm attributed to a $^2T_{1g} \rightarrow {}^2T_{2g}$ transition in aqueous solution.[28]

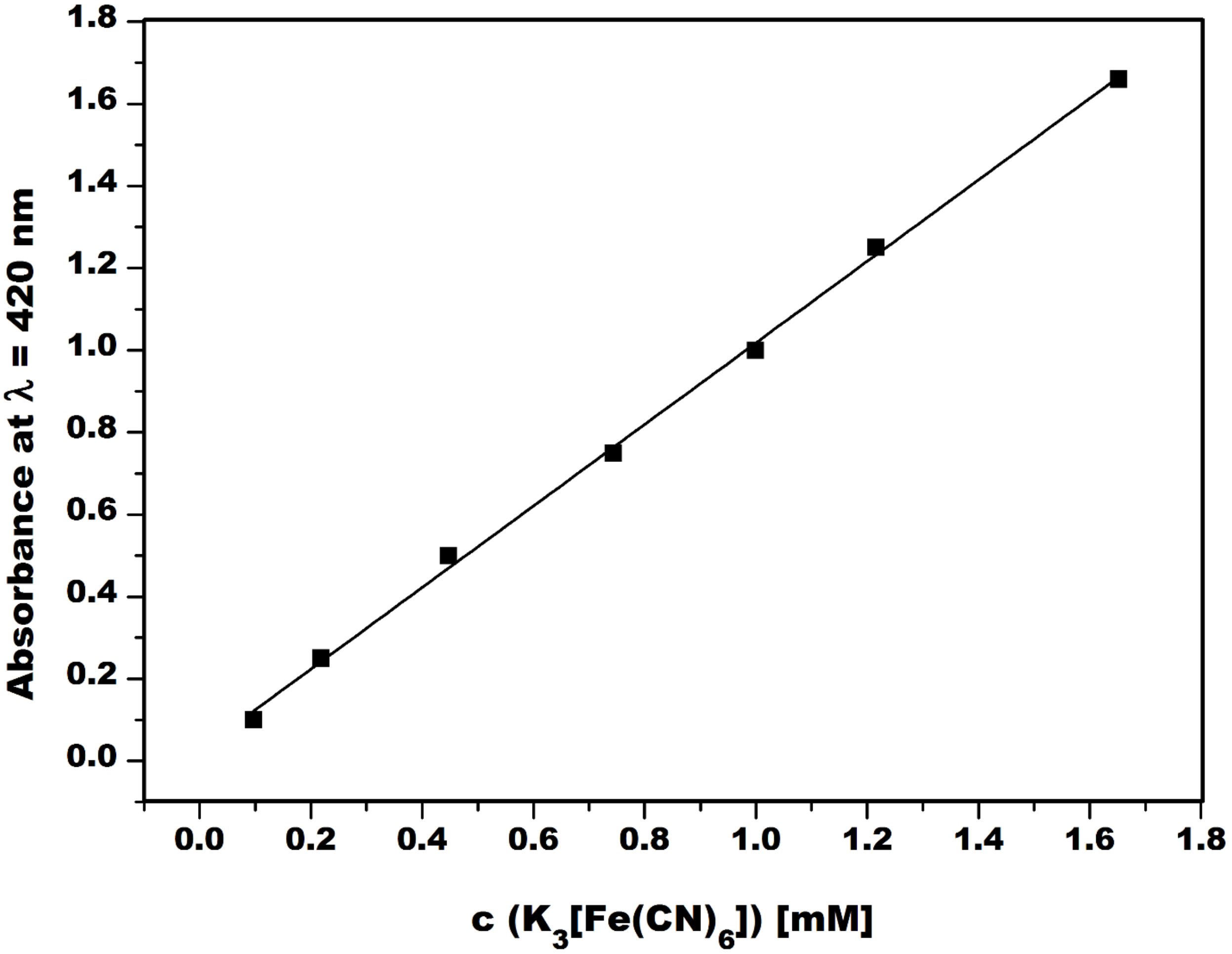

Figure S20. Standard curve obtained by plotting the absorbance values measured at 420 nm versus concentration of the corresponding $[Fe^{III}(CN)_6]^{3-}$ stock solutions (**Figure S19**) (R^2 = 0.99).

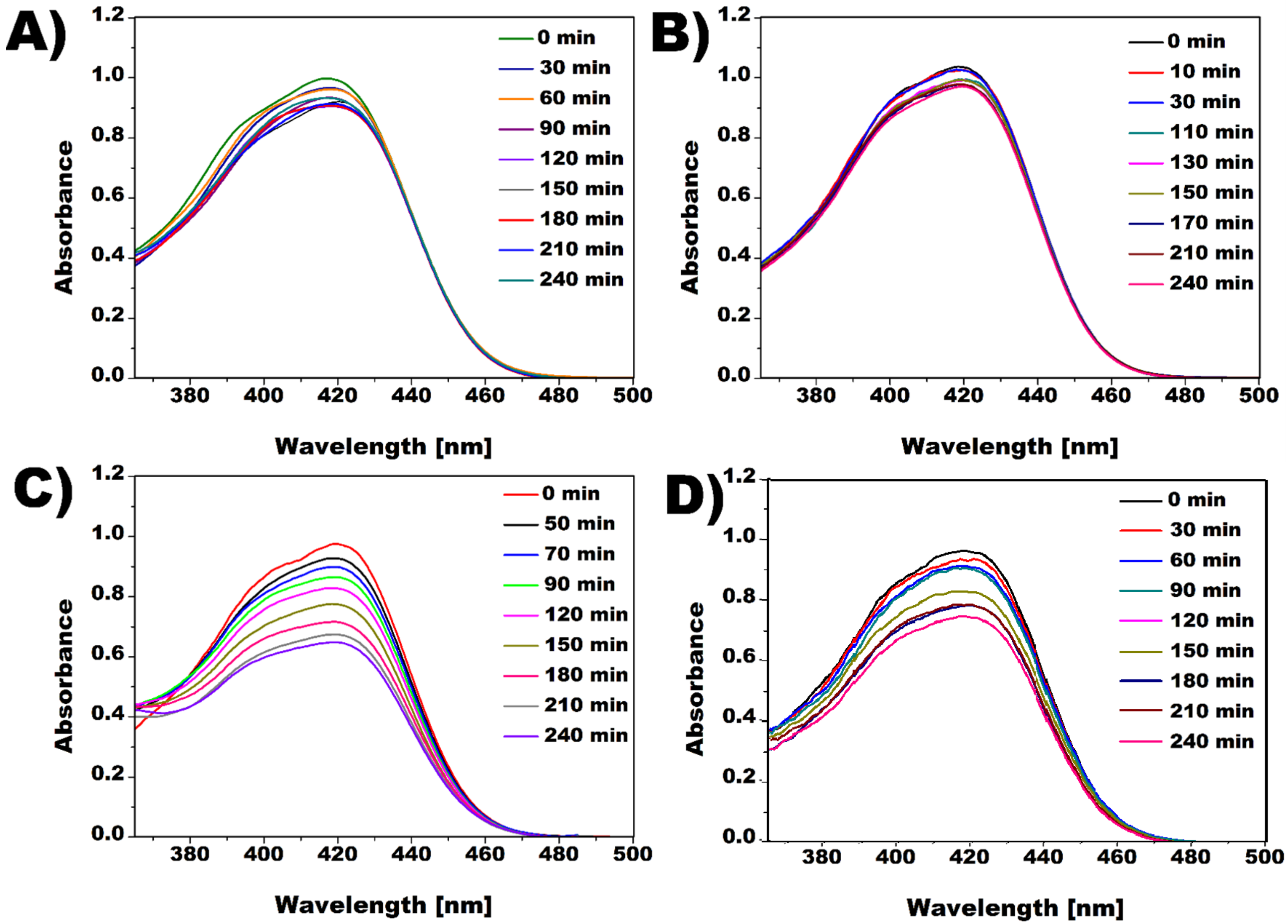

Figure S21. Time-dependent UV/Vis spectra of 1 mM $[Fe^{III}(CN)_6]^{3-}$ at 55°C in the presence of **A)** 8.7 mM $Na_2S_2O_3$ lacking catalyst, **B)** 80 µM **{Sb_3W_{43}}** lacking $Na_2S_2O_3$, **C)** 80 µM **{Sb_3W_{43}}** and 8.7 mM $Na_2S_2O_3$, **D)** 80 µM **{Bi_3W_{43}}** and 8.7 mM $Na_2S_2O_3$.

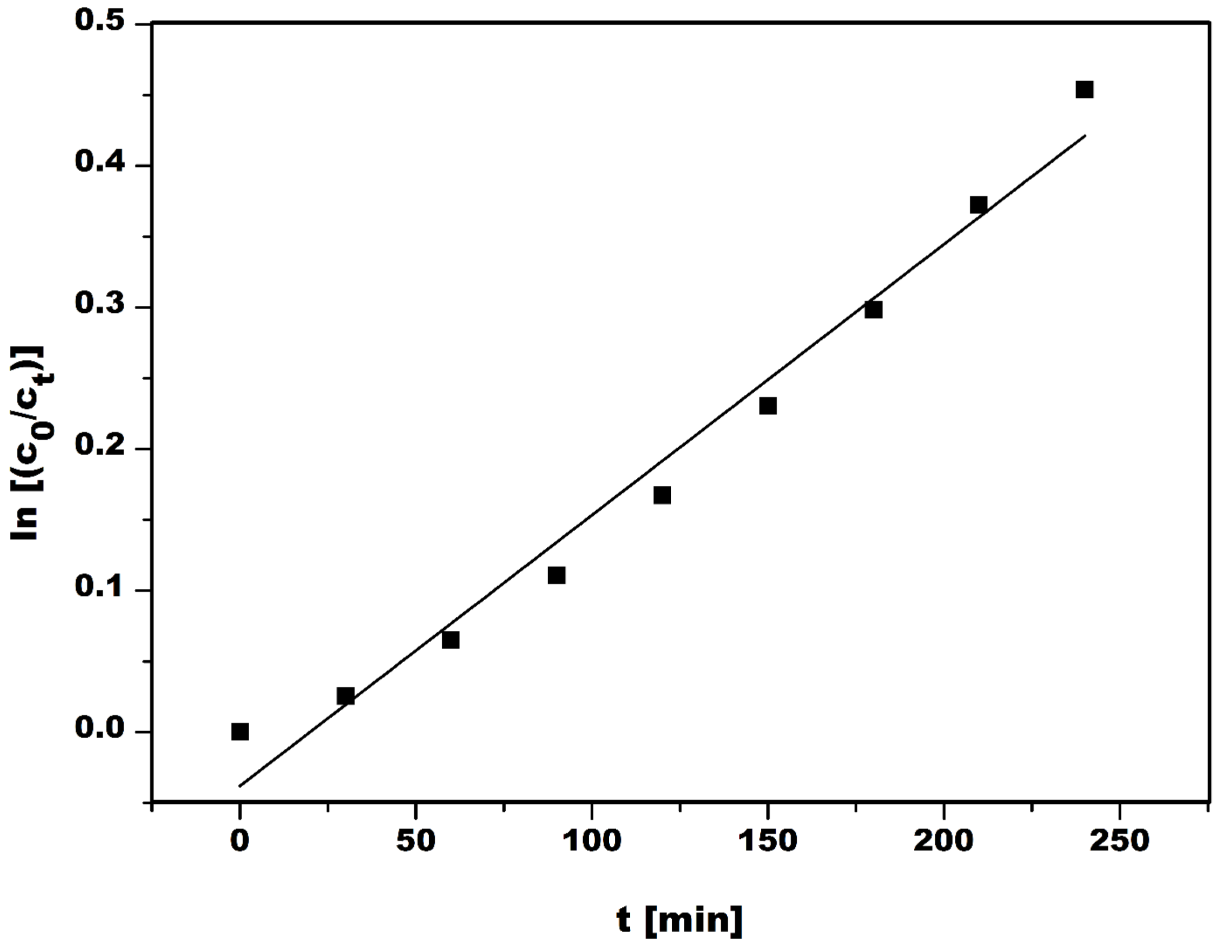

Figure S22. Rate curve for the reduction of $K_3[Fe^{III}(CN)_6]$ catalyzed by **{Sb_3W_{43}}** with c_0 and c_t being the $K_3[Fe^{III}(CN)_6]$ concentrations at given times 0, 30, 60, 90, 120, and 150, 180, 210, 240 min. The slope of the linear fit (R^2 = 0.97): ln [(c_0/c_t)] = 19.1 (± 1.1) × 10^{-4} x - 0.038 with x being the reaction time in min gives the rate constant k = 19.1 (± 1.1) × 10^{-4} min^{-1}.

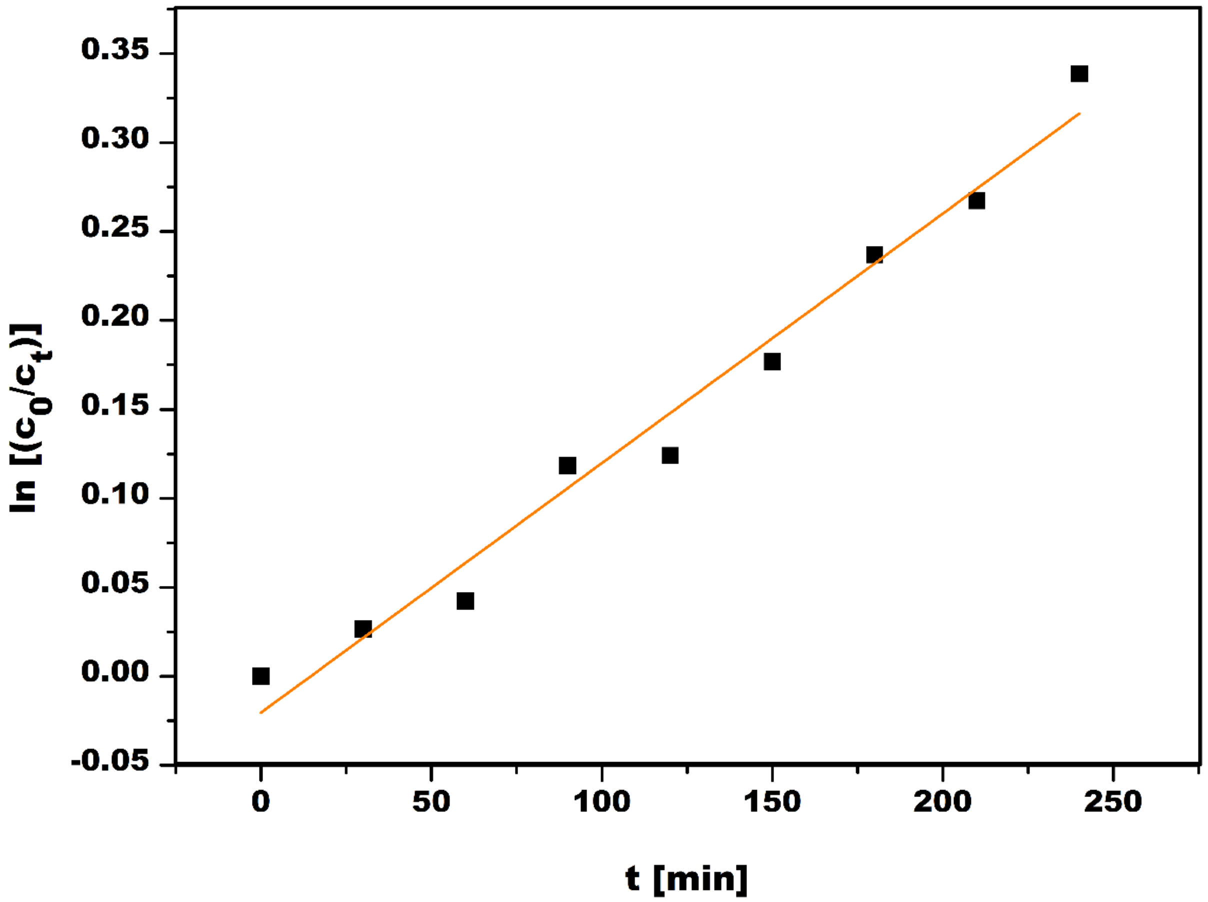

Figure S23. Rate curve for the reduction of $K_3[Fe^{III}(CN)_6]$ catalyzed by **{Bi$_3$W$_{43}$}** with c_0 and c_t being the $K_3[Fe^{III}(CN)_6]$ concentrations at given times 0, 30, 60, 90, 120, and 150, 180, 210, 240 min. The slope of the linear fit (R^2 = 0.97): ln [(c_0/c_t)] = 14.1 (± 0.8) × 10^{-4} x - 0.021 with x being the reaction time in min gives the rate constant k = 14.1 (± 0.8) × 10^{-4} min^{-1}.

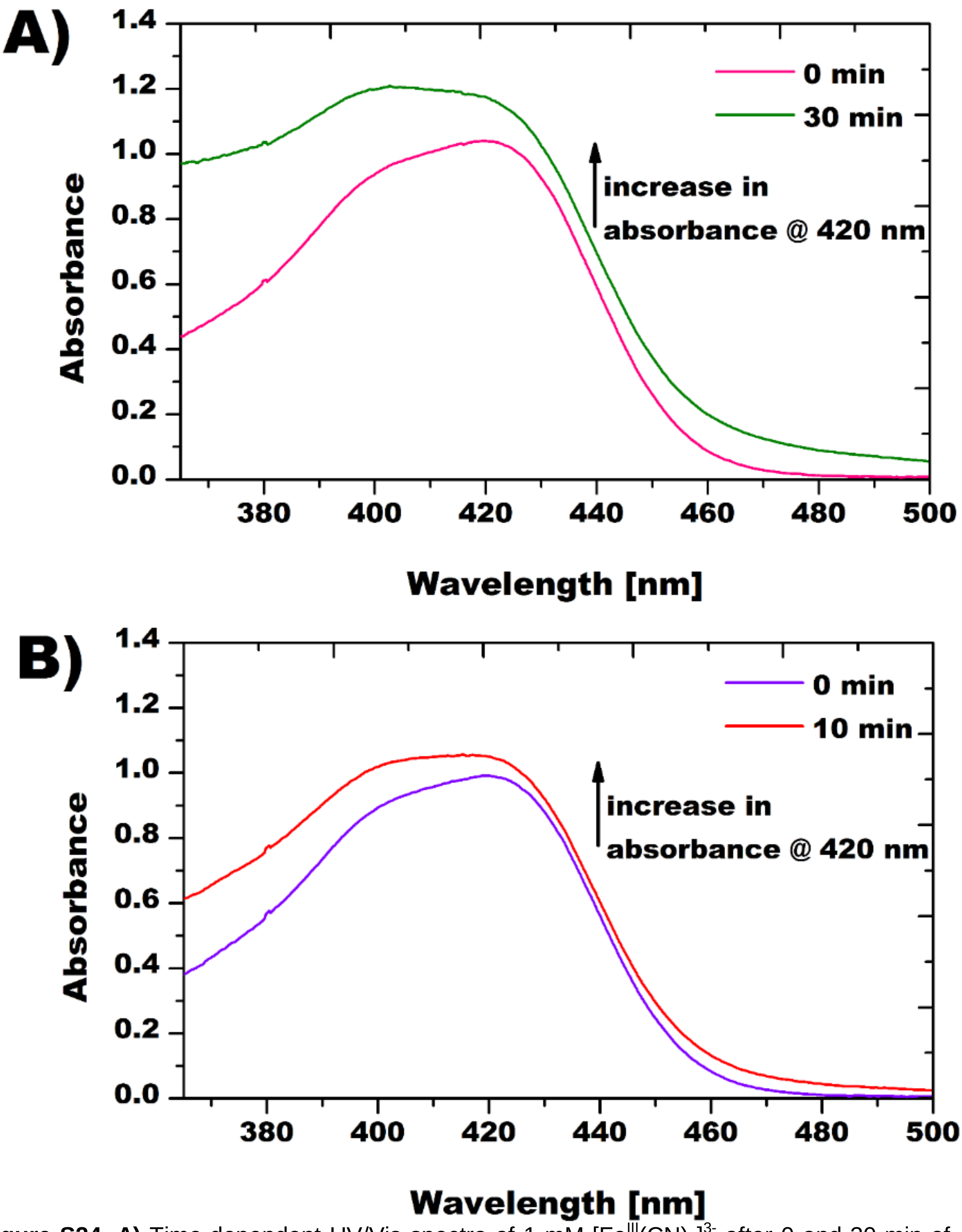

Figure S24. A) Time-dependent UV/Vis spectra of 1 mM $[Fe^{III}(CN)_6]^{3-}$ after 0 and 30 min of incubation at 55°C in the presence of 80 µM **{$Co_3Sb_3W_{42}$}** and 8.7 mM $Na_2S_2O_3$ **B)** after 0 and 10 min of incubation at 55°C in the presence of 80 µM $CoCl_2 \cdot 6H_2O$ and 8.7 mM $Na_2S_2O_3$. In both cases, an increase of the absorption at 420 nm could be observed indicating leeching of free Co(II) upon decomposition of **{$Co_3Sb_3W_{42}$}**.

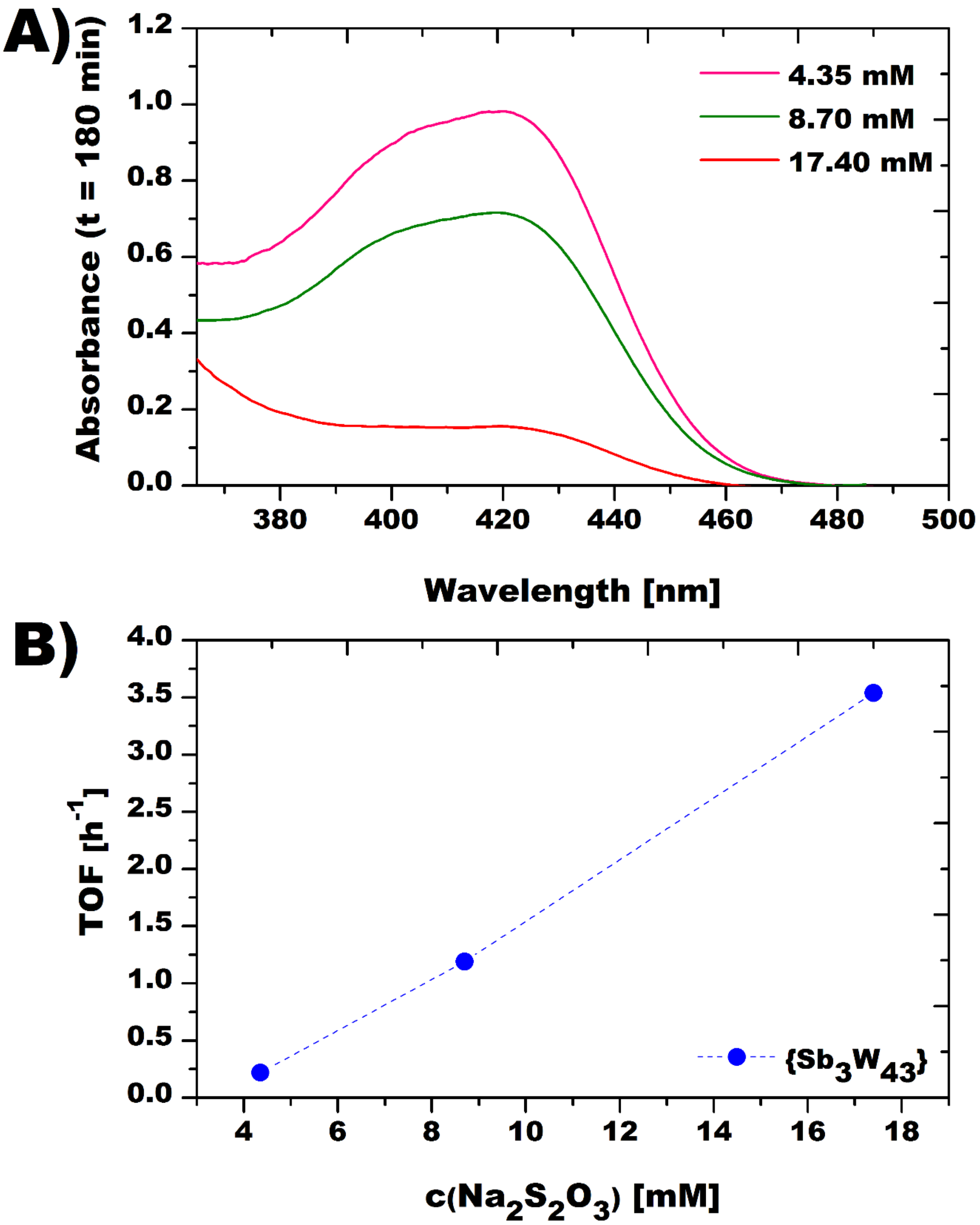

Figure S25. A) UV/Vis spectra of 1 mM $[Fe^{III}(CN)_6]^{3-}$ after 180 min of incubation at 55°C in the presence of 80 µM **{Sb_3W_{43}}** and varying concentrations of $Na_2S_2O_3$ (4.35 – 14.7 mM) resulting in **B)** different turnover frequency (TOF) values determined at t = 180 min.

9. Post-Catalysis

9.1. Post-catalytic POM-precipitation for subsequent analysis with ATR-IR

The catalytic reaction was carried out with 500 µM of **Sb_3W_{43}** or **Bi_3W_{43}**, 1 mM $[Fe^{III}(CN)_6]^{3-}$ and 8.7 mM $Na_2S_2O_3$ in 1.5 mL H_2O (pH = 6.8 via HCl [1 M]) to ensure sufficient amounts for post-analysis. After incubation at 55°C for 40 h to ensure complete reduction of the $[Fe^{III}(CN)_6]^{3-}$ substrate, solid cesium chloride was added to the reaction mixture resulting in the immediate formation of precipitates. The precipitates were centrifuged at 2500 rpm for 5 min and completeness of the precipitation was insured by adding cesium chloride to the supernatant and by performing a subsequent reloading experiment (see section 9.2). The precipitates were air dried and displayed to IR-spectroscopic analysis (**Figures S26, S27**).

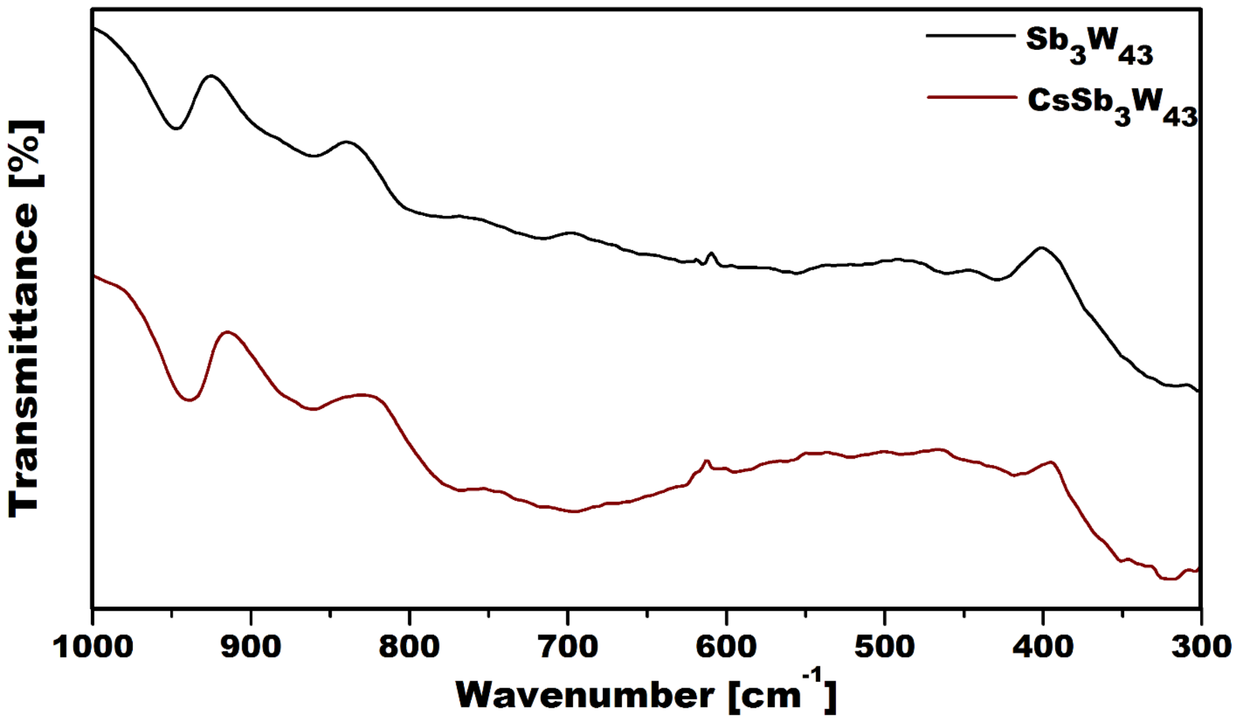

Figure S26. IR-spectra showing the tungsten fingerprint area from 300 - 1000 cm^{-1} of **{Sb_3W_{43}}**, (black), and the precipitated polyanion catalyst by addition of cesium chloride after reaction with $[Fe^{III}(CN)_6]^{3-}$ for 40 h at 55°C, pH = 6.8 **{$CsSb_3W_{43}$}** (wine red).

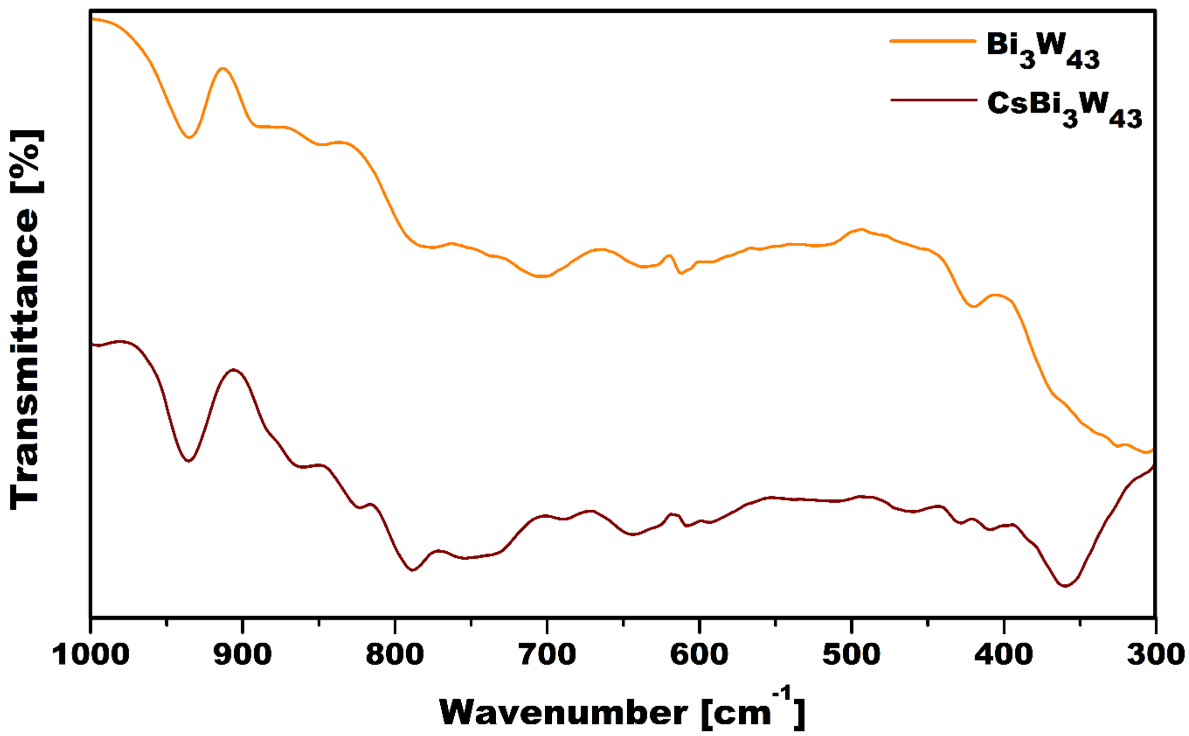

Figure S27. IR-spectra showing the tungsten fingerprint area from 300 - 1000 cm^{-1} of **{Bi_3W_{43}}**, (orange), and the precipitated polyanion catalyst by addition of cesium chloride after reaction with $[Fe^{III}(CN)_6]^{3-}$ for 40 h at 55°C, pH = 6.8 **{$CsBi_3W_{43}$}** (wine red).

9.2. Reloading experiment of the reaction mixture after post-catalytic POM-precipitation

Subsequent addition of 1 mM $[Fe^{III}(CN)_6]^{3-}$ to the remaining supernatants obtained by the procedure described in section 9.1 and incubation at 55°C to initiate a second reaction cycle showed negligent conversion of $[Fe^{III}(CN)_6]^{3-}$ (~8 % according to the UV/Vis spectra in **Figures S28** and **S29**), which suggests complete removal of the corresponding POT catalyst upon addition of CsCl.

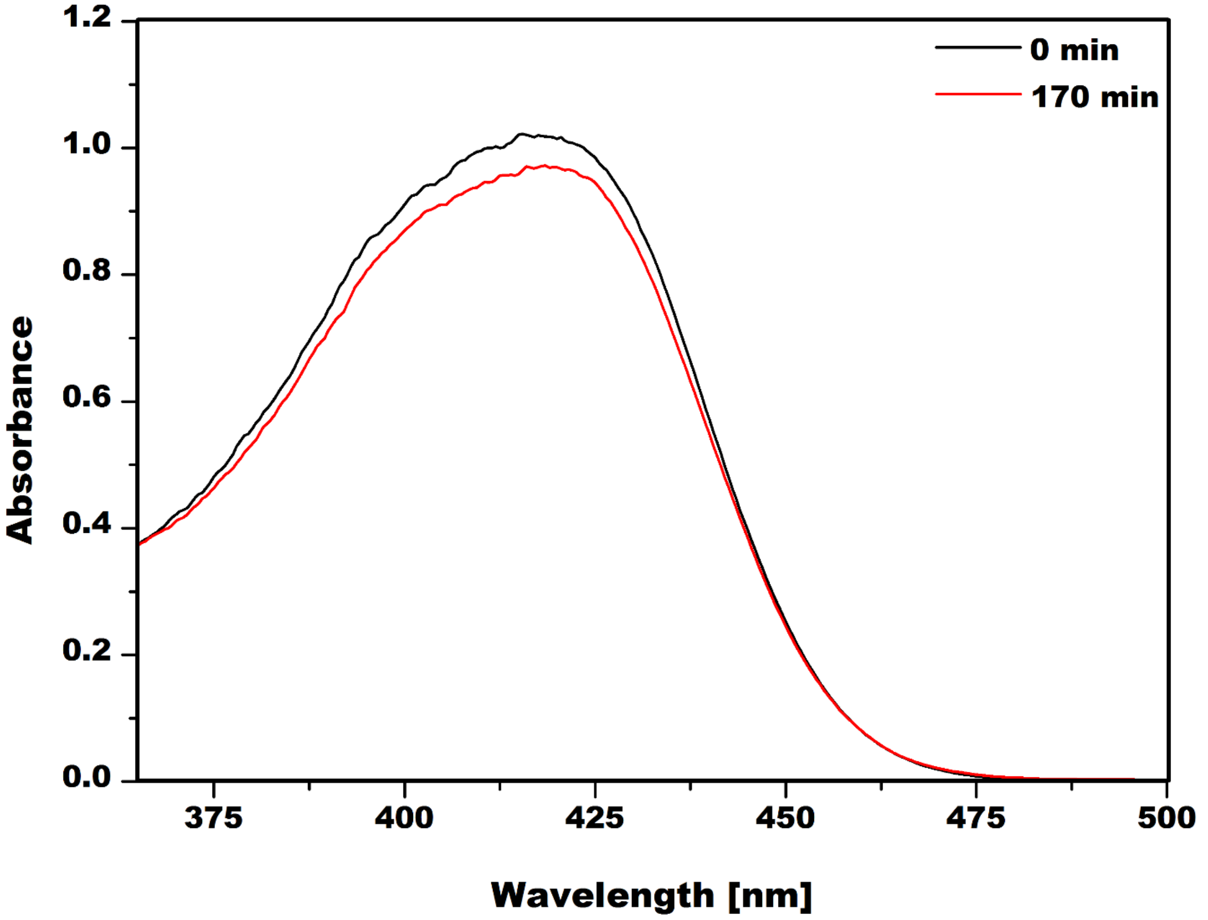

Figure S28. UV/Vis spectra of the reaction mixture ([8.7 mM] $Na_2S_2O_3$, [1 mM] $[Fe^{III}(CN)_6]^{3-}$, [80 µM] **$\{Sb_3W_{43}\}$**) after precipitation of the polyanion using cesium chloride (**Figure S26**) and reloading with $[Fe^{III}(CN)_6]^{3-}$. Incubation at 55°C shows a significant drop of $[Fe^{III}(CN)_6]^{3-}$ conversion indicating complete removal of the catalyst upon precipitation.

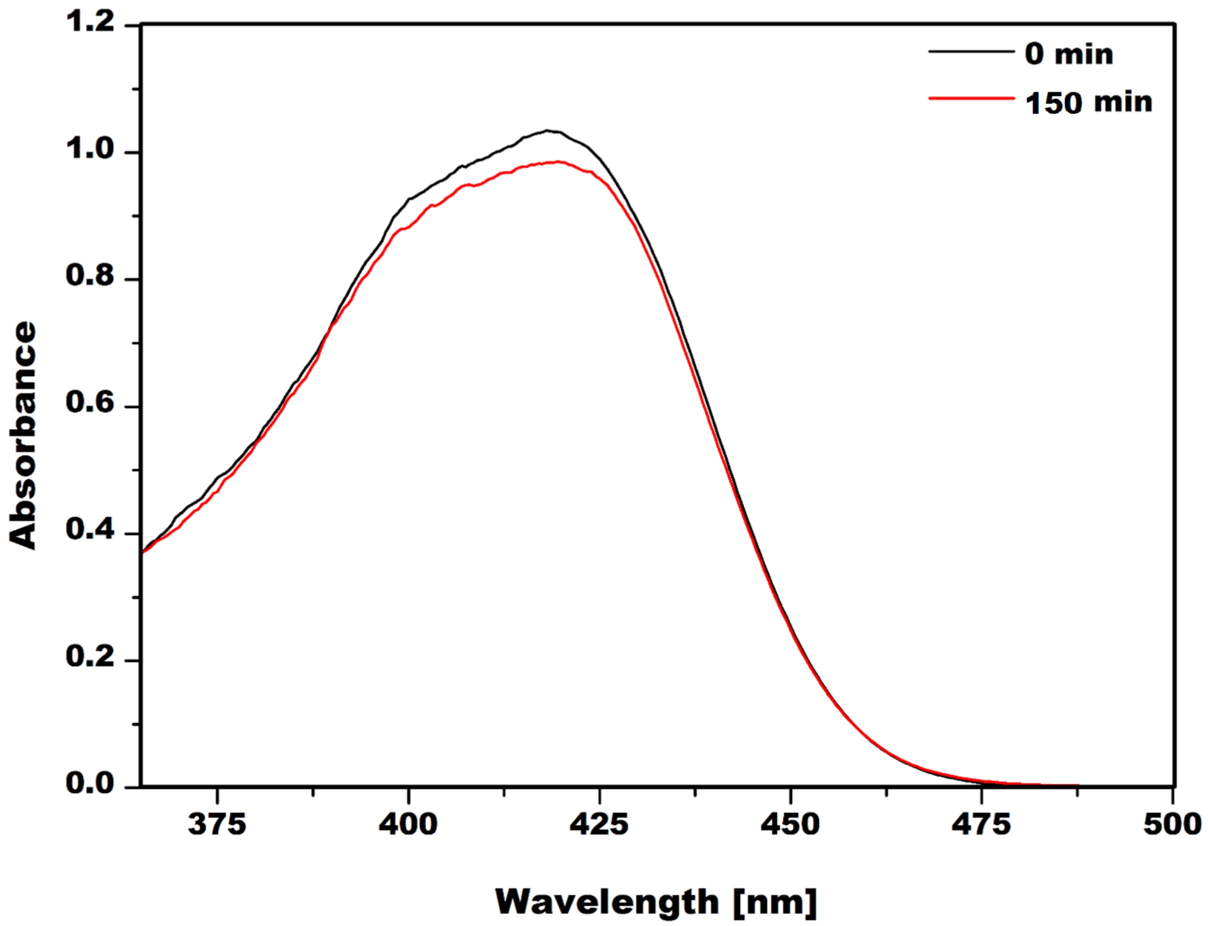

Figure S29. UV/Vis spectra of the reaction mixture ([8.7 mM] $Na_2S_2O_3$, [1 mM] $[Fe^{III}(CN)_6]^{3-}$, [80 µM] **{Bi_3W_{43}}**) after precipitation of the polyanion using cesium chloride (**Figure S27**) and reloading with $[Fe^{III}(CN)_6]^{3-}$. Incubation at 55°C shows a significant drop of $[Fe^{III}(CN)_6]^{3-}$ conversion indicating complete removal of the catalyst upon precipitation.

9.3. Recyclability of {Sb_3W_{43}} and {Bi_3W_{43}}

To investigate the recyclability of **Sb_3W_{43}** and **Bi_3W_{43}**, a stock solution containing 80 µM of the corresponding POT, 1 mM $[Fe^{III}(CN)_6]^{3-}$ and 8.7 mM $Na_2S_2O_3$ in 1.5 mL H_2O was incubated at 55°C for 72 h and the complete conversion (~98% based on the absorption at 420 nm) of the $[Fe^{III}(CN)_6]^{3-}$ substrate was shown by UV/Vis spectroscopy. Consecutively, 5 µL of a freshly prepared solution containing [62 mM] $[Fe^{III}(CN)_6]^{3-}$ were added to 300 µL of the incubated stock solution followed by addition of 5 µL of a 539.4 mM solution containing $Na_2S_2O_3$ to yield 310 µL of a reloaded reaction mixture with 1 mM $[Fe^{III}(CN)_6]^{3-}$ and ~8.7 mM $Na_2S_2O_3$ final concentrations **(Figures S30, S31)**.

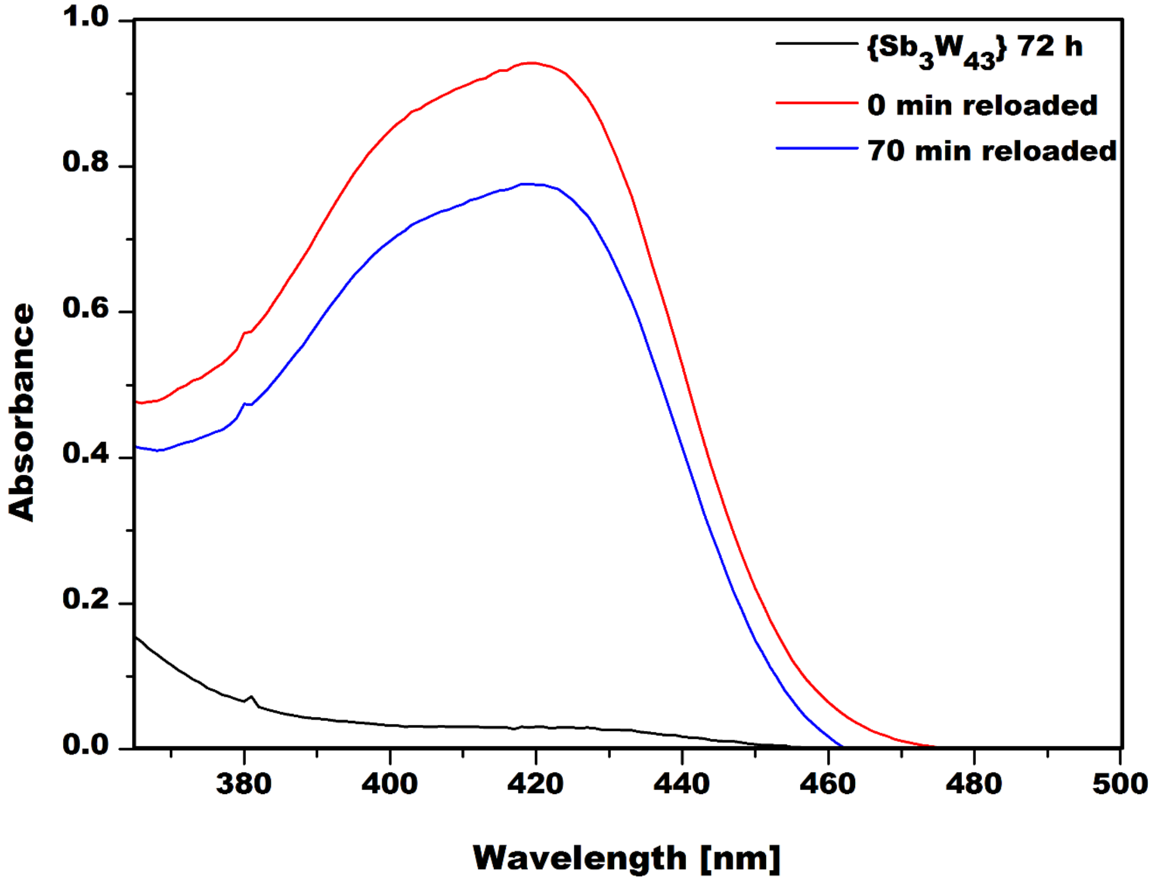

Figure S30. UV/Vis spectra of the reaction mixture ([8.7 mM] $Na_2S_2O_3$, [1 mM] $[Fe^{III}(CN)_6]^{3-}$, [80 µM] **{Sb_3W_{43}}**) after 72 h of incubation at 55°C showing 98% conversion of $[Fe^{III}(CN)_6]^{3-}$. Reloading of the reaction mixture with $Na_2S_2O_3$ and $[Fe^{III}(CN)_6]^{3-}$ and consecutive incubation at 55°C leads to conversion of the substrate indicated by a decreasing peak at 420 nm thereby highlighting the recyclability of **{Sb_3W_{43}}**. Note that the slightly higher TOF values are attributed to the presence of additionally added reducing agent in the second cycle.

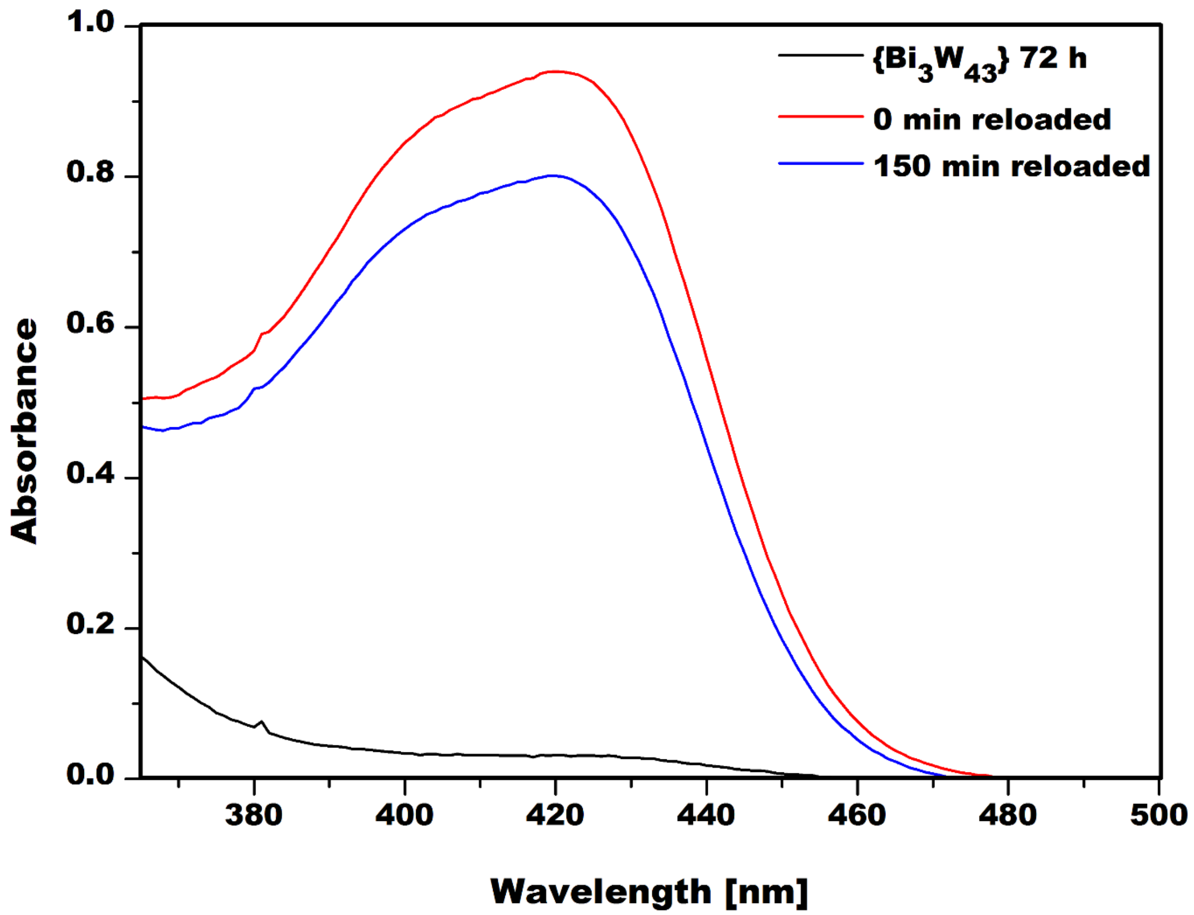

Figure S31. UV/Vis spectra of the reaction mixture ([8.7 mM] $Na_2S_2O_3$, [1 mM] $[Fe^{III}(CN)_6]^{3-}$, [80 µM] **{Bi_3W_{43}}**) after 72 h of incubation at 55°C showing 98% conversion of $[Fe^{III}(CN)_6]^{3-}$. Reloading of the reaction mixture with $Na_2S_2O_3$ and $[Fe^{III}(CN)_6]^{3-}$ and consecutive incubation at 55°C leads to conversion of the substrate indicated by a decreasing peak at 420 nm thereby highlighting the recyclability of **{Bi_3W_{43}}**. Note that the slightly higher TOF values are attributed to the presence of additionally added reducing agent in the second cycle.

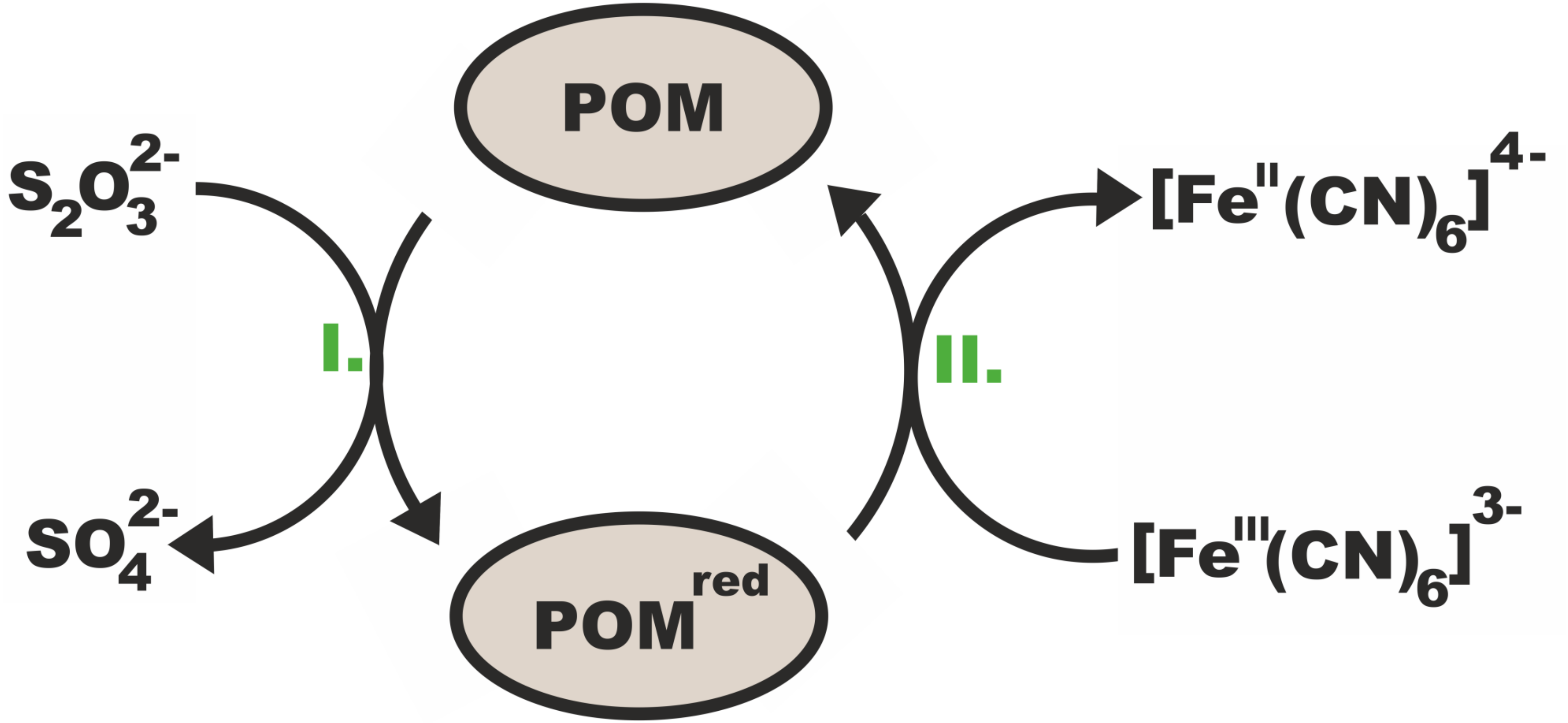

Scheme S1. Schematic representation of the mechanism proposed for the POM catalyzed reduction reaction of $[Fe^{III}(CN)_6]^{3-}$ to $[Fe^{II}(CN)_6]^{4-}$ upon oxidation of $S_2O_3^{2-}$ to SO_4^{2-}. The electron transfer reaction is catalyzed by **{Bi_3W_{43}}** or **{Sb_3W_{43}}**, which is temporarily reduced by $S_2O_3^{2-}$ (I.) and subsequently acts as a reducing agent to reduce $[Fe^{III}(CN)_6]^{3-}$ to $[Fe^{II}(CN)_6]^{4-}$ (II.).

10. References

1 Bösing, M.; Loose, I.; Pohlmann, H.; Krebs, B. New Strategies for the Generation of Large Heteropolymetalate Clusters: The β- *B*- SbW_9 Fragment as a Multifunctional Unit. *Chem.-Eur. J.*, **1997**, *3*, 1232–1237.

2 Mbomekalle, I.-M.; Lu, Y. W.; Keita, B.; Nadjo, L. Simple, High Yield and Reagent-Saving Synthesis of Pure α-$K_6P_2W_{18}O_{62}\cdot 14H_2O$. *Inorg. Chem. Commun.* **2004**, *7*, 86–90.

3 Bruker SAINT v7.68A Copyright © 2005-2016 Bruker AXS.

4 Sheldrick, G. M. SADABS University of Göttingen, Germany (1996).

5 Sheldrick, G. M. (1996) SHELXS. University of Göttingen, Germany.

6 G. M. Sheldrick, (1996) SHELXL. University of Göttingen, Germany.

7 Dolomanov, O. V.; Bourhis, L. J.; Gildea, R. J.; Howard, J. A. K.; Puschmann, H. OLEX2. *J. Appl. Cryst.* **2009**, *42*, 339-341.

8 Huebschle, C. B.; Sheldrick, G. M.; Dittrich, B. ShelXle: a Qt graphical user interface for SHELXL. *J. Appl. Cryst*. **2011**, *44*, 1281-1284.

9 Schäffer, C.; Merca, A.; Bögge, H.; Todea, A. M.; Kistler, M. L.; Liu, T.; Thouvenot, R.; Gouzerh, P.; Müller, A. Unprecedented and Differently Applicable Pentagonal Units in a Dynamic Library: A Keplerate of the Type $\{(W)W_5\}_{12}\{Mo_2\}_{30}$. *Angew. Chem. Int. Ed.* **2009**, *48*, 149-153.

10 Leclerc - Laronze, N.; Marrot, J.; Thouvenot, R.; Cadot, E. Structural Link between Giant Molybdenum Oxide Based Ions and Derived Keggin Structure: Modular Assemblies Based on the $[BW_{11}O_{39}]^{9-}$ Ion and Pentagonal {M'M$_5$} Units (M' = W; M = Mo, W). *Angew. Chem. Int. Ed.* **2009**, *48*, 4986 –4989.

11 Todea, A. M.; Merca, A.; Bögge, H.; Glaser, T.; Pigga, J. M.; Langston, M. L. K.; Liu, T.; Prozorov, R.; Luban, M.; Schröder, C.; Casey, W. H.; Müller, A. Porous Capsules $\{(M)M_5\}_{12}Fe^{III}_{30}$ (M=Mo^{VI}, W^{VI}): Sphere Surface Supramolecular Chemistry with 20 Ammonium Ions, Related Solution Properties, and Tuning of Magnetic Exchange Interactions. *Angew. Chem. Int. Ed.* **2009**, *48*, 514 – 519.

12 De la Oliva, A. R.; Sans, V.; Miras, H. N.; Yan, J.; Zang, H.; Richmond, C. J.; Long, D. - L.; Cronin, L. Assembly of a Gigantic Polyoxometalate Cluster $\{W_{200}Co_8O_{660}\}$ in a Networked Reactor System. *Angew. Chem. Int. Ed.* **2012**, *51*, 12759 – 12762.

13 Zhan, C.; Cameron, J. M.; Gao, J.; Purcell, J. W.; Long, D. - L.; Cronin, L. Time-Resolved Assembly of Cluster-in-Cluster $\{Ag_{12}\}$-in-$\{W_{76}\}$ Polyoxometalates under Supramolecular Control. *Angew. Chem. Int. Ed.* **2014**, *53*, 10362 –10366.

14 Zhan, C. - H.; Winter, R. S.; Zheng, Q.; Yan, J.; Cameron, J. M.; Long, D. - L.; Cronin, L. Assembly of Tungsten-Oxide-Based Pentagonal Motifs in Solution Leads to Nanoscale $\{W_{48}\}$, $\{W_{56}\}$, and $\{W_{92}\}$ Polyoxometalate Clusters. *Angew. Chem. Int. Ed.* **2015**, *54*, 14308 –14312.

15 Yan, J.; Gao, J.; Long, D. - L.; Miras, H. N.; Cronin, L. Self-Assembly of a Nanosized, Saddle-Shaped, Solution-Stable Polyoxometalate Anion Built from

Pentagonal Building Blocks: $[H_{34}W_{119}Se_8Fe_2O_{420}]^{54-}$. *J. Am. Chem. Soc.* **2010**, *132*, 11410–11411.

16 Gao, J.; Yan, J.; Beeg, S.; Long, D. - L.; Cronin, L. One-Pot versus Sequential Reactions in the Self-Assembly of Gigantic Nanoscale Polyoxotungstates. *J. Am. Chem. Soc.* **2013**, *135*, 1796−1805.

17 Zhang, Y. - Y.; Liu, S. - X.; Yu, C. - J.; Tang, Q.; Liang, D. - D.; Zhang, C. - D.; Ma, F. - J.; Li, S. - J.; Zhang, W.; Tan, R. - K. A new Dawson-like tungstoantimonate related to $[Sb^{V}W_{18}O_{60}(OH)_2]^{9-}$. *Inorg. Chem. Commun.* **2010**, *13*, 1418-1420.

18 Park, K. M.; Ozawa, Y.; Lee, U. Crystal Structure of Hexapotassium Undecahydrogen Tetratungsto Hexaantimonate(Ⅴ) Tetrahydrate. *J. Korean Chem. Soc.*, **1994**, *38*, 359-365.

19 Lee, U.; Sasaki, Y. Crystal Structure of Pentapotassium Disodium Hexatungstoantimonate(V) Dodecahydrate, $K_5Na_2[SbW_6O_{24}]\cdot 12H_2O$. *Bull Korean Chem Soc*. **1987**, *8*, 1-3, b) Naruke, H.; Yamase, T. Structure of a Photoluminescent Polyoxotungstoantimonate. *Acta Cryst*. **1992**, *48*, 597-599.

20 Jeannin, Y. Novel vacant heteropolytungstate with a X_2W_{21} skeleton derived from the polytungstate $[H_6X_2W_{22}O_{76}]^{8-}$ (X = Sb, Bi). *CR CHIM*. **2004**, *7*, 1235-1240.

21 Xin, X.; Ma, Y.; Hou, L.; Wang, Y.; Xue, X.; Lin, J.; Han, Z. Krebs-Type $\{M_2(WO_2)_2[B\text{-}\beta\text{-}SbW_9O_{33}]_2\}^{n-}$ (M = Sb^{III}, (WO_3)) Tungstoantimonate Possessing Unique Pseudo-Seesaw Sb−O Structure. *Inorg. Chem.* **2019**, *58*, 9567−9571.

22 Senevirathna, D. C.; Werrett, M. V.; Kubeil, M.; Stephan, H.; Andrews P. C. Synthesis, structural characterisation, and cytotoxicity studies of Bi, W, and Mo containing homo- and hetero-bimetallic polyoxometalates. *Dalton Trans.*, **2019**, *48*, 15962-15969.

23 Patrut, A.; Bögge, H.; Forizs, E.; Rusu, D.; Lowy, D. A.; Margineanu, D.; Naumescu, A. *Rev. Roum. Chim.* **2010**, *55*, 865-870.

24 Sun, C. – Y.; Liu, S. – X.; Wang, C. – L.; Xie, L. – H.; Zhang, C. – D; Gao, B.; Wang, E. – B. Reactions of trivacant lone-pair-containing tungstobismutate and electrochemical behaviors of its sandwich-type products. *J. Coord. Chem.*, **2007**, *60*, 567-579.

25 Xu, Z. – H.; Wang, X. – L.; Li, Y. – G.; Wang, E. – B.; Qin, C.; Si; Y. – L. A novel bismuth ion-bridged chainlike assembly from paradodecatungstate $[H_2W_{12}O_{42}]^{10-}$ anions: $(NH_4)_7[Bi(H_2W_{12}O_{42})]$ • $20H_2O$. *Inorg. Chem. Commun.*, **2007**, *10*, 276-278.

26 Rodewald, D.; Jeannin, Y. Etude cristallographique et par résonance magnétique nucléaire du bismuthooctadécatungstate $Na_7[H_2BiW_{18}O_{60}]$ • $24H_2O$ Crystallographic and nuclear magnetic resonance study of bismuthooctadecatungstate $Na_7[H_2BiW_{18}O_{60}]$ • $24H_2O$. *C. R. Acad. Sci.*, **1999**, *2*, 63-67.

27 Ozawa, Y.; Sasaki, Y. Synthesis and Crystal Structure of $[(CH_3)_4N]_6[H_3BiW_{18}O_{60}]$. *Chem. Lett.* **1987**, *16*, 923–926.

28 Ayers, J. B.; Waggoner, W. H. Synthesis and properties of two series of heavy metal hexacyanoferrates. *J. Inorg. Nucl. Chem.*, **1971**, *33*, 721-733.
